# Homocysteine thiolactone and other sulfur-containing amino acid metabolites are associated with fibrin clot properties and the risk of ischemic stroke

**DOI:** 10.1038/s41598-024-60706-2

**Published:** 2024-05-16

**Authors:** Marta Sikora, Ewa Bretes, Joanna Perła-Kaján, Olga Utyro, Kamila Borowczyk, Justyna Piechocka, Rafał Głowacki, Izabela Wojtasz, Radosław Kaźmierski, Hieronim Jakubowski

**Affiliations:** 1 European Centre for Bioinformatics and Genomics, Institute of Bioorganic Chemistry, 61-704 Poznań, Poland; 2 Department of Biochemistry and Biotechnology, Poznań University of Life Sciences, 60-632 Poznań, Poland; 3 Faculty of Chemistry, Department of Environmental Chemistry, University of Łódź, 90-236 Łódź, Poland; 4 Medicover, 61-131 Poznań, Poland; 5 Department of Neurology, Collegium Medicum, University of Zielona Góra, 65-046 Zielona Góra, Poland; 6 Department of Neurology, Poznań University of Medical Sciences, 60-355 Poznań, Poland; 7 Department of Microbiology, Biochemistry and Molecular Genetics, Rutgers-New Jersey Medical School, International Center for Public Health, 225 Warren Street, Newark, NJ 07103 USA

**Keywords:** Homocysteine thiolactone, Sulfur amino acids, Fibrin clot properties, Stroke, Risk factors, Biochemistry

## Abstract

Homocysteine (Hcy) and Hcy-thiolactone (HTL) affect fibrin clot properties and are linked to cardiovascular disease. Factors that influence fibrin clot properties and stroke are not fully understood. To study sulfur-containing amino acid metabolites, fibrin clot lysis time (CLT) and maximum absorbance (Abs_max_) in relation to stroke, we analyzed plasma and urine from 191 stroke patients (45.0% women, age 68 ± 12 years) and 291 healthy individuals (59.7% women, age 50 ± 17 years). Plasma and urinary levels of sulfur-containing amino acid metabolites and fibrin clot properties were significantly different in stroke patients compared to healthy individuals. Fibrin CLT correlated with fibrin Abs_max_ in healthy males (R^2^ = 0.439, *P* = 0.000), females (R^2^ = 0.245, *P* = 0.000), female stroke patients (R^2^ = 0.187, *P* = 0.000), but not in male stroke patients (R^2^ = 0.008, *P* = ns). Fibrin CLT correlated with age in healthy females but not males while fibrin Abs_max_ correlated with age in both sexes; these correlations were absent in stroke patients. In multiple regression analysis in stroke patients, plasma (p)CysGly, pMet, and *MTHFR A1298C* polymorphism were associated with fibrin Abs_max_, while urinary (u)HTL, uCysGly, and pCysGly were significantly associated with fibrin CLT. In healthy individuals, uHTL and uGSH were significantly associated with fibrin Abs_max_, while pGSH, and *CBS T833C 844ins68* polymorphism were associated with fibrin CLT. In logistic regression, uHTL, uHcy, pCysGly, pGSH, *MTHFR C677T* polymorphism, and Abs_max_ were independently associated with stroke. Our findings suggest that HTL and other sulfur-containing amino acid metabolites influence fibrin clot properties and the risk of stroke.

## Introduction

Stroke is one of the leading causes of morbidity and mortality in the world^1^. Overall stroke burden increased across the globe in both men and women of all ages^2^. Many traditional risk factors for stroke are known and include hypertension, diabetes, obesity, dyslipidemia, glycemia, etc.^3^. Ischemic stroke causes an injury in the brain that is mediated by thrombotic or embolic events originating from a cardiac source or periphery^4^. Thrombus formation involves the generation of a fibrin mesh scaffold that involves complex interactions between components of the coagulation cascade^5^. Accumulating evidence suggests that fibrin clot properties are associated with the development and progression of venous and thromboembolic disease^6^. For example, altered fibrin clot structure and function, reflected in increased maximum absorbance (Abs_max_), and longer clot lysis time (CLT), have been seen in cryptogenic stroke patients^7^. Another study reported that prolonged CLT did not affect the risk of stroke in young women whereas decreased CLT increases the risk^8^. Finding factors that affect fibrin clot properties is important in assessing the risk of stroke and in the development of preventive and treatment strategies^9^.

Elevated plasma (p) total homocysteine (pHcy) is a non-traditional risk factor for stroke^10^. Changes in pHcy levels are known to affect levels of other sulfur-containing amino acid metabolites^11^. However, how the sulfur-containing amino acid metabolites are related to stroke and how they affect fibrin clot structure and function in stroke patients was not known. In an earlier study with a large cohort of coronary artery disease (CAD) patients (n ~ 2000), we found that urinary Hcy-thiolactone (uHTL)^12,13^ and plasma cysteine (pCys)^13^ were associated with fibrin clot properties and predicted myocardial infarction (MI). These findings have led to a hypothesis that an increased risk of stroke is mediated by the unfavorable effects of sulfur-containing amino acid metabolites on fibrin clot properties. The present study was designed to test this hypothesis by examining the associations of sulfur-containing amino acid metabolites with fibrin clot properties and stroke. We also studied determinants of CLT and Abs_max_ in stroke patients and healthy individuals.

## Results

### Sex-specific effects of stroke on fibrin clot properties

The study population included 191 ischemic stroke patients, 45.0% were female, and the mean age was 68 ± 12 years. Healthy controls included 291 individuals, 59.4% were female, and the mean age was 50 ± 17 years. Descriptive statistics of all variables analyzed in the present study are shown in Supplementary Table S2 online.

**Figure 1:**
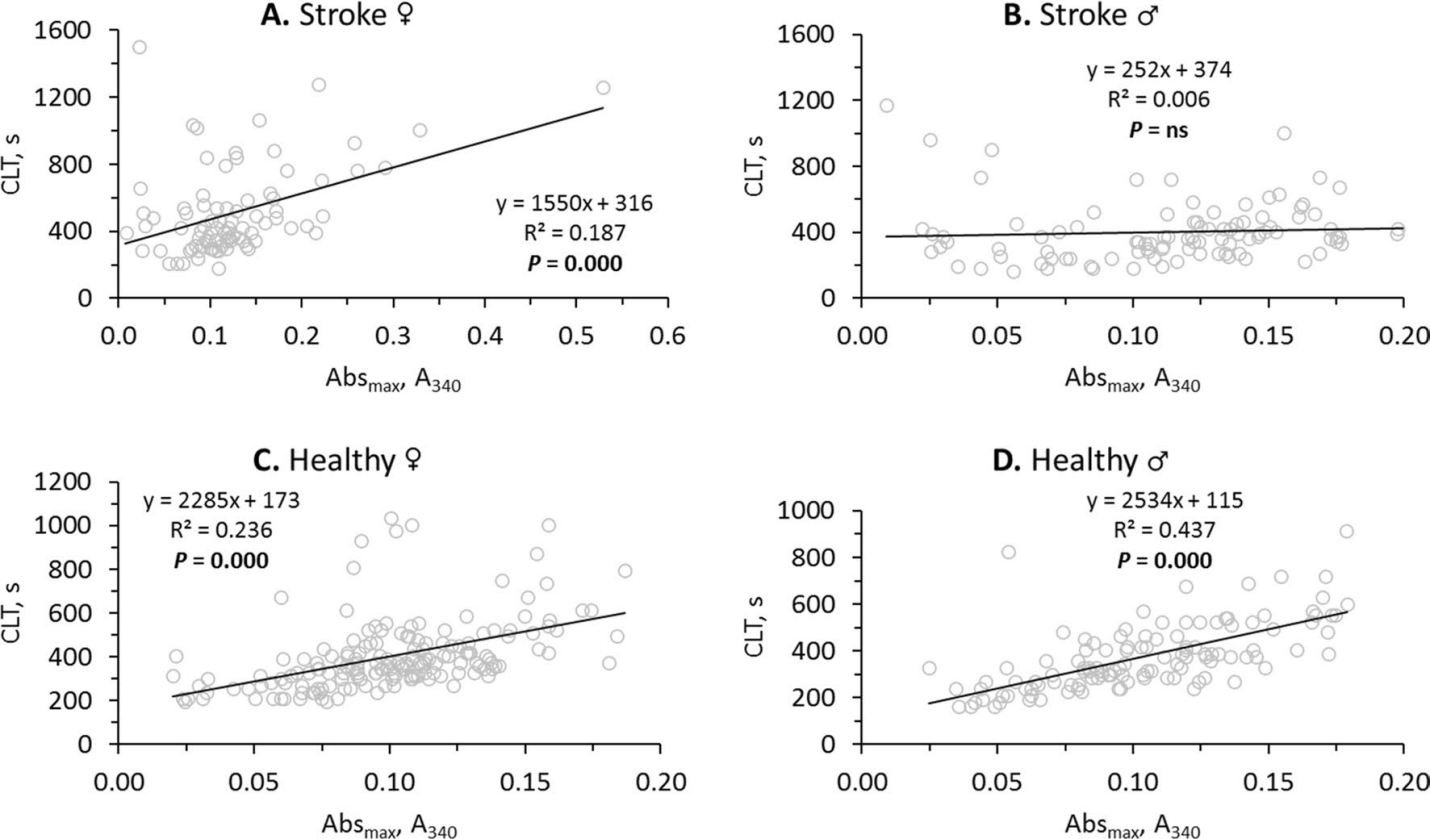
Sex affects relationships between fibrin CLT and Abs_max_ in ischemic stroke patients (**A**,**B**) but not in healthy individuals (**C**,**D**).

Analysis of fibrin clot formation and lysis (see Supplementary Fig. S1) showed that the kinetics of these processes were changed and the correlations between the involved variables were attenuated in stroke patients compared to healthy controls (Supplementary Table S2). For example, the correlation between fibrin CLT and Abs_max_ was reduced to 0.29 in stroke patients from 0.55 in healthy individuals (Supplementary Table S2). This reduction can be traced to different effects of stroke on CLT and Abs_max_ in men and women. Specifically, fibrin CLT and Abs_max_ were strongly correlated in female stroke patients (R^2^ = 0.187, *P* = 0.000; Fig. 1A). In contrast, in male stroke patients, there was no correlation between CLT and Abs_max_ (R^2^ = 0.006, *P* = ns; Fig. 1B). However, CLT and Abs_max_ were strongly correlated both in healthy females (R^2^ = 0.236, *P* = 0.000; Fig. 1C) and healthy males (R^2^ = 0.437, *P* = 0.000; Fig. 1D).

Fibrin CLT was significantly longer in female stroke patients compared to healthy women (510 ± 264 *vs*. 400 ± 161 s, *P* = 0.000). In contrast, fibrin CLT was not significantly changed in male stroke patients compared to healthy males (405 ± 177 *vs*. 377 ± 143 s, *P* = 0.181 (Table 1). Sex significantly affected fibrin CLT in stroke patients (longer in women than in men: 510 ± 264 *vs*. 405 ± 177 s, *P* = 0.001) but not in healthy individuals (401 ± 161 *vs*. 377 ± 143 s, *P* = 0.199) (Table 1).

**Table 1:** Fibrin clot lysis time (CLT), maximum absorbance (Abs_max_), and other variables in stroke patients and healthy controls stratified by sex. Significant values are in bold.

Variable	Stroke patients (*n* = 191)			Healthy controls (*n* = 291)			*P*_strokeF_	*P*_strokeM_
	Women (n = 85)	Men (n = 106)	*P*_sex_	Women (n = 173)	Men (n = 118)	*P*_sex_		
CLT, s	508 ± 264	405 ± 177	**0.001**	401 ± 161	377 ± 143	0.187	**0.000**	0.181
Abs_max_, A_340_	0.124 ± 0.075	0.122 ± 0.056	0.844	0.100 ± 0.035	0.104 ± 0.037	0.339	**0.000**	**0.003**
uHTL, nM	36 ± 95	24 ± 71	0.299	40 ± 86	66 ± 77	**0.010**	0.729	**0.000**
uHcy, μM	9.2 ± 9.2	11.4 ± 10.9	0.131	4.8 ± 3.7	8.9 ± 5.7	**0.000**	**0.000**	**0.031**
uCys, μM	265 ± 207	316 ± 190	0.072	181 ± 124	280 ± 132	**0.000**	**0.000**	0.099
uCysGly, μM	10.2 ± 7.2	10.9 ± 7.7	0.522	8.45 ± 5.60	11.5 ± 5.6	**0.000**	**0.029**	0.495
uGSH, μM	3.5 ± 3.4	3.3 ± 3.2	0.578	5.0 ± 5.5	5.6 ± 5.4	0.347	**0.021**	**0.000**
Urinary creatinine, mM	9.8 ± 6.9	12.8 ± 7.1	**0.003**	10 ± 7	17 ± 9	**0.000**	0.682	**0.000**
Plasma Met, μM	39 ± 17	42 ± 19	0.277	41 ± 19	45 ± 20	0.065	0.608	0.316
Plasma Hcy, μM	7.5 ± 3.6	7.3 ± 3.0	0.578	5.4 ± 1.7	6.9 ± 2.8	**0.000**	**0.000**	0.334
Plasma Cys, μM	272 ± 63	256 ± 58	0.058	222 ± 63	235 ± 74	0.113	**0.000**	**0.020**
Plasma CysGly, μM	17 ± 9	17 ± 9	0.997	18 ± 11	22 ± 13	**0.004**	0.551	**0.002**
Plasma GSH, μM	3.3 ± 1.7	3.3 ± 1.8	0.802	3.9 ± 3.2	4.6 ± 3.0	0.179	0.073	**0.000**
Plasma sulfur, mM	28.4 ± 4.3	28.7 ± 3.5	0.491	28.6 ± 2.0	29.0 ± 1.9	0.082	0.492	0.427
Plasma creatinine, μM	76 ± 21	94 ± 40	**0.000**	64 ± 9	81 ± 12	**0.000**	**0.000**	**0.001**
GFR, mL/min/1.73 m^2^	71.9 ± 18.2	76.2 ± 16.8	0.079	85.6 ± 8.1	87.6 ± 7.1	**0.034**	**0.000**	**0.000**
Age, years	71 ± 11	66 ± 12	**0.007**	53 ± 16	46 ± 17	**0.000**	**0.000**	**0.000**

Fibrin Abs_max_ was significantly elevated both in female (0.124 ± 0.074 *vs*. 0.100 ± 0.035 A_340_, *P* = 0.000) and male stroke patients (0.122 ± 0.056 *vs*. 0.103 ± 0.037 A_340_, *P* = 0.003) (Table 1). Sex did not affect Abs_max_ in stroke patients (0.124 ± 0.074 A_340_ in females *vs*. 0.122 ± 0.056 A_340_ in males, *P* = 0.844) nor in healthy individuals (0.100 ± 0.035 A_340_ in females *vs*. 0.103 ± 0.037 A_340_ in males, *P* = 0. 399) (Table 1).

### Sex-specific effects of stroke on levels of sulfur-containing metabolites

Levels of uHTL were significantly reduced in male (but not female, *P* = 0.729) stroke patients compared to healthy controls (24 ± 71 *vs*. 66 ± 77 nM,* P* = 0.000), while levels of uCys and uCysGly were elevated in female (but not male, *P* = 0.099 and *P* = 0.495, respectively) stroke patients (uCys: 265 ± 207 vs. 181 ± 124 μM, *P* = 000; uCysGly: 10.2 ± 7.2 vs. 8.4 ± 5.5 μM, *P* = 0.024). Levels of uHcy were significantly elevated in stroke patients compared to healthy controls (female: 9.2 ± 9.2 *vs*. 4.8 ± 3.7 μM, *P* = 0.000; male: 11.4 ± 10.9 *vs*. 8.9 ± 5.7 μM, *P* = 0.031). Levels of uGSH were significantly reduced in stroke patients (female: 3.5 ± 3.4 *vs*. 5.0 ± 5.5 μM, *P* = 0.021; male: 3.3 ± 3.2 *vs*. 5.6 ± 5.4 μM, *P* = 0.000) (Table 1).

Female stroke patients (but not male) had significantly elevated pHcy compared to healthy controls (7.5 ± 3.6 *vs*. 5.4 ± 1.7 μM, *P* = 0.000) while pCys was elevated both in female (272 ± 63 *vs*. 222 ± 63 μM, *P* = 0.000) and male (256 ± 58 vs. 235 ± 74 μM, *P* = 0.020) stroke patients. Levels of pCysGly (17 ± 9 *vs*. 22 ± 13 μM, *P* = 0.002) and pGSH (3.3 ± 1.8 *vs*. 4.6 ± 3.0 μM, *P* = 0.000) were significantly reduced in male (but not in female, *P* = 0.551 and *P* = 0.073, respectively) stroke patients. However, plasma methionine (pMet) was not changed in female (*P* = 0.608) or male (*P* = 0.316) stroke patients compared to healthy individuals (Table 1).

Changes in creatinine levels associated with ischemic stroke were also sex specific. For example, urinary creatinine was significantly reduced in male stroke patients compared to healthy males (12.8 ± 7.1 vs. 17 ± 9 mM,* P* = 0.000) but was not changed in female stroke patients compared to healthy females (9.8 ± 6.9 *vs*. 10.1 ± 6.7 mM,* P* = 0.682). However, plasma creatinine was significantly elevated in ischemic stroke patients, both female and male (female: 76 ± 21 vs. 64 ± 9 μM,* P* = 0.000; male: 94 ± 40 *vs*. 81 ± 12 μM, *P* = 0.001) (Table 1).

### Sex-specific effects of age on fibrin clot properties

**Figure 2:**
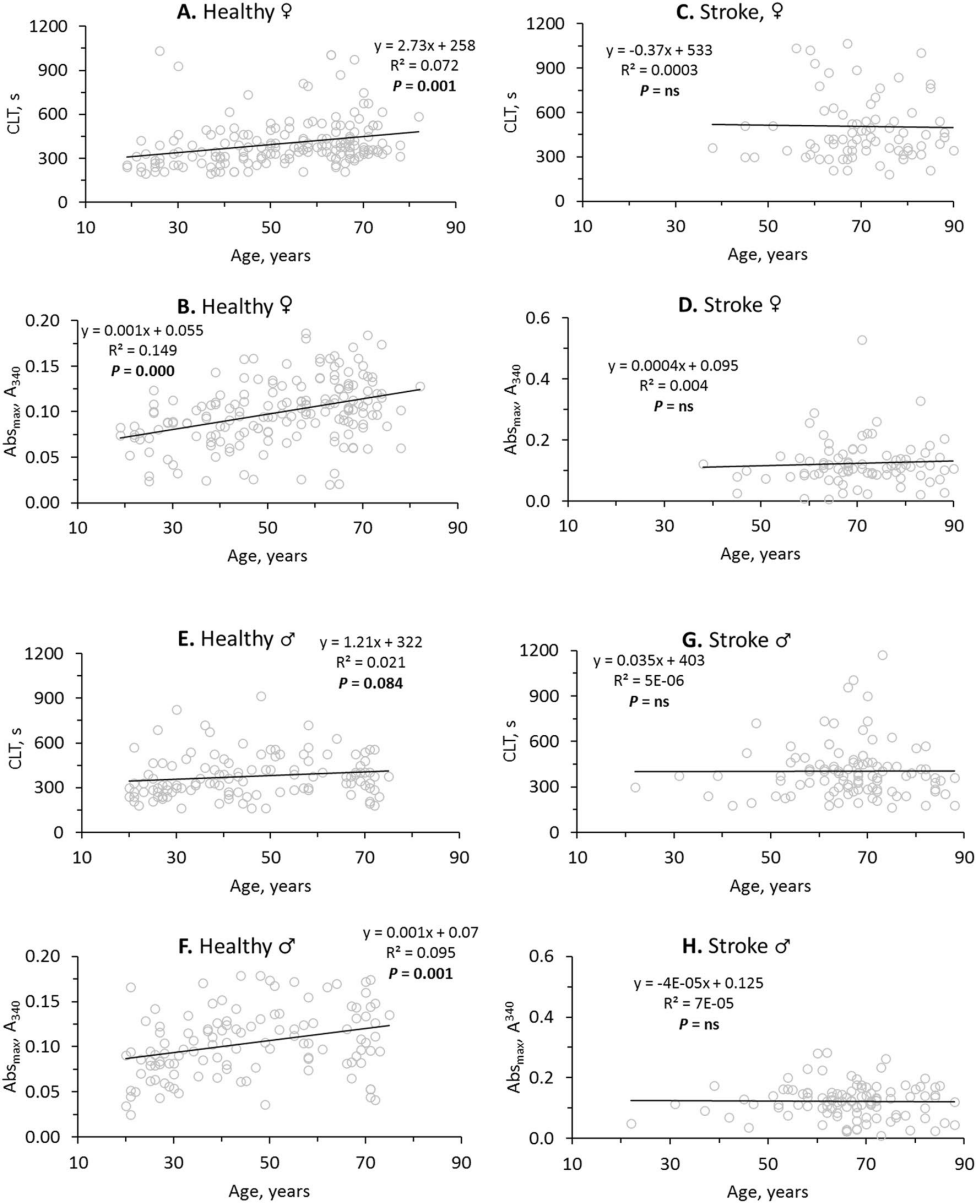
Stroke abrogates the influence of age on fibrin clot properties. Pearsons’s correlations between fibrin CLT (**A**,**C**,**E**,**G**), Abs_max_ (**B**,**D**,**F**,**H**) and age in healthy individuals (**A**,**B**,**E**,**F**) and ischemic stroke patients (**C**,**D**,**G**,**H**) are shown. Higher values of CLT and Abs_max_ indicate lower susceptibility to lysis and worse clot structure, respectively.

In healthy females, fibrin CLT (R^2^ = 0.072, *P* = 0.001; Fig. 2A) and fibrin Abs_max_ (R^2^ = 0.149, *P* = 0.000; Fig. 2B) significantly increased with age. In contrast, age did not affect CLT (R^2^ = 0.0003, *P* = ns; Fig. 2C) nor Abs_max_ (R^2^ = 0.004, *P* = ns; Fig. 2D) in female stroke patients.

In contrast to healthy females, CLT was not affected by age in healthy males (R^2^ = 0.021, *P* = 0.084; Fig. 2E). However, like in healthy females, significant increases of Abs_max_ with age were seen in healthy males (R^2^ = 0.095, *P* = 0.001; Fig. 2F). Like in female stroke patients, age did not affect CLT (R^2^ = 5E−06, *P* = ns; Fig. 2G) and Abs_max_ (R^2^ = 7E−05, *P* = ns; Fig. 2H) in male stroke patients.

In healthy females the intercept of the trendline at the CLT axis was 258 s and the CLT was increasing at a rate of 2.73 s/year (Fig. 2A) while in female stroke patients the intercept was elevated to 533 s and there was no significant change in CLT with age (Fig. 2C). This shows that in young female stroke patients CLT has already reached the maximal high value (Fig. 2C), characteristic of CLT in older healthy females (Fig. 2A). Similarly, in young female (Fig. 2D) and male (Fig. 2H) stroke patients Abs_max_ has already reached the maximal high value, characteristic of CLT in older healthy females (Fig. 2B) and males (Fig. 2F). These findings suggest prothrombotic fibrin clot properties in young stroke patients like those of older healthy individuals.

### Sulfur-containing amino acid metabolite levels affect fibrin clot properties in healthy individuals but not in ischemic stroke patients

**Figure 3:**
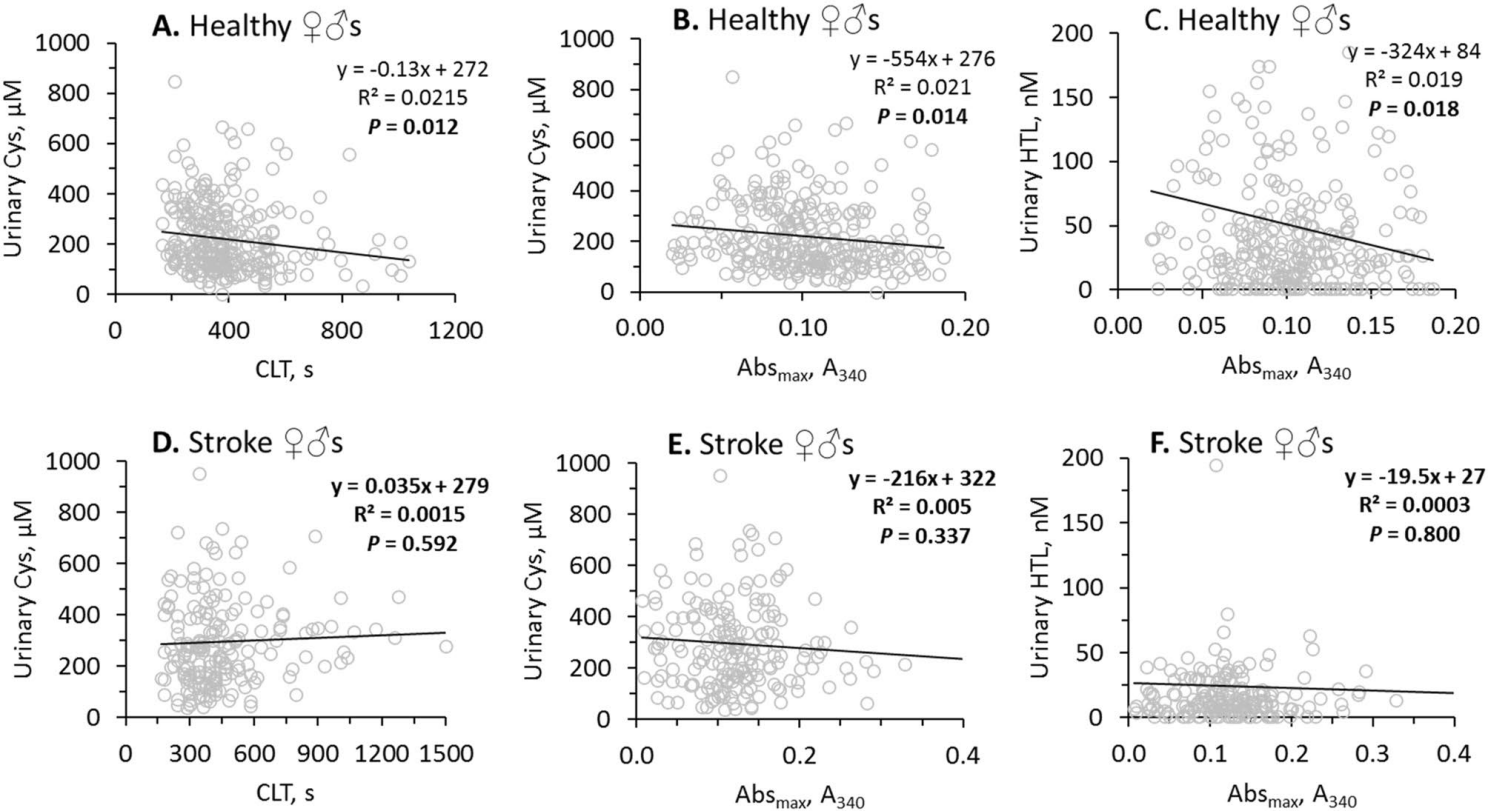
Pearsons’s correlations between urinary Cys, HTL and fibrin CLT, Abs_max_ are abrogated by stroke. Higher values of CLT and Abs_max_ indicate lower susceptibility to lysis and worse clot structure, respectively.

In healthy individuals, urinary sulfur-containing amino acid metabolites were negatively correlated with fibrin CLT and Abs_max_ (Table 2). Specifically, uCys was significantly correlated with CLT (R^2^ = 0.0215, *P* = 0.012; Fig. 3A) and Abs_max_ (R^2^ = 0.021, *P* = 0.014; Fig. 3B) while uHTL was significantly correlated only with Abs_max_ (R^2^ = 0.019, *P* = 0.018; Fig. 3C). Weaker correlations were observed for uHcy with CLT (R^2^ = 0.013, *P* = 0.054) and Abs_max_ (R^2^ = 0.016, *P* = 0.043), for uCysGly with CLT (R^2^ = 0.018, *P* = 0.023) and Abs_max_ (R^2^ = 0.015, *P* = 0.038), and for uGSH with Abs_max_ (R^2^ = 0.013, *P* = 0.055) (see Supplementary Figure S2).

**Table 2:** *P* values for correlations between fibrin clot properties vs. urinary (u) sulfur-containing amino acid metabolites, uCreatinine, and for correlations between urinary (u) sulfur-containing amino acid metabolites themselves in ischemic stroke patients and healthy individuals.

	Fibrin clot		Sulfur-containing amino acid metabolites					
Variable	CLT	Abs_max_,	uHcy	uCys	uCysGly	uGSH	uHTL	uCreatinine
Healthy individuals, *P* values*								
CLT		0.000( +)	**0.051(−)**	**0.010(−)**	**0.021(−)**	ns	ns	**0.001(−)**
Abs_max_	0.000( +)		**0.030(−)**	**0.008(−)**	**0.022(−)**	**0.039(−)**	**0.014(−)**	**0.007(−)**
uHcy	**0.051(−)**	**0.030(−)**		0.000( +)	0.000( +)	0.000( +)	**0.000( +)**	0.000( +)
uCys	**0.010(−)**	**0.008(−)**			0.000( +)	0.000( +)	**0.000( +)**	0.000( +)
uCysGly	**0.021(−)**	**0.022(−)**				0.000( +)	**0.000( +)**	0.000( +)
uGSH	ns	**0.039(−)**					**0.000( +)**	0.000( +)
uHTL	ns	**0.014(−)**						**0.000**( +)
Stroke patients, *P* values*								
CLT		0.000( +)	**ns**	**ns**	**ns**	ns	ns	**ns**
Abs_max_	0.000( +)		**ns**	**ns**	**ns**	**ns**	**ns**	**ns**
uHcy	**ns**	**ns**		0.000( +)	0.000( +)	0.000( +)	**ns**	0.000( +)
uCys	**ns**	**ns**			0.000( +)	0.000( +)	**ns**	0.000( +)
uCysGly	**ns**	**ns**				0.000( +)	**ns**	0.000( +)
uGSH	ns	**ns**					**ns**	0.000( +)
uHTL	ns	**ns**						**ns**

*P values affected by stroke are highlighted in bold. Symbols (**−**) and ( +) show negative and positive correlations, respectively.

In contrast, in ischemic stroke patients there were no correlations of uCys with CLT (Fig. 3D) and Abs_max_ (Fig. 3E), and uHTL with Abs_max_ (Fig. 3F). There were also no correlations of uHcy, uCysGly and uGSH with CLT and/or Abs_max_ in ischemic stroke patients (see Supplementary Figure S2). Furthermore, correlations between uHTL and other urinary sulfur-containing amino acid metabolites seen in healthy individuals were also absent in ischemic stroke patients (Table 2). However, correlations between urinary creatinine and other urinary sulfur-containing amino acid metabolites seen in healthy individuals were also seen in ischemic stroke patients (Table 2).

These findings suggest that efficient urinary excretion of sulfur-containing amino acid metabolites in healthy individuals can contribute to favorable clot properties, i.e. structure that is less compact and more susceptible to lysis.

In contrast to urinary sulfur-containing amino acid metabolites, plasma sulfur-containing amino acid metabolites (pHcy, pCys, pCysGly, pMet, and pGSH) were not associated with fibrin CLT nor Abs_max_ in healthy individuals. These plasma metabolites were also not associated with fibrin CLT nor Abs_max_ in ischemic stroke patients (see Supplementary Table S3). These findings suggest that the urinary sulfur-containing amino acid metabolites might be better predictors of ischemic stroke risk than the plasma metabolites.

While urinary creatinine was associated with CLT and Abs_max_, plasma creatinine was associated with neither (see Supplementary Table S3).

### Relationships of sulfur-containing amino acid metabolites with age and GFR in ischemic stroke patients and healthy individuals

#### Age

In healthy individuals, urinary sulfur-containing amino acid metabolites, uHcy (R^2^ = 0.101, *P* = 0.000), uCys (R^2^ = 0.143, *P* = 0.000), uCysGly (R^2^ = 0.118, *P* = 0.000), and uHTL (R^2^ = 0.018, *P* = 0.021) significantly decreased with age while uGSH levels were not affected by age (see Supplementary Figure S3).

In contrast, in ischemic stroke patients age did not significantly affect levels of uCysGly (R^2^ = 0.030, *P* = 0.092) and uHTL (R^2^ = 0.010, *P* = 0.171). However, uHcy (R^2^ = 0.013, *P* = 0.002) and uCys (R^2^ = 0.045, *P* = 0.005) remained negatively associated with age in ischemic stroke patients. Like in healthy individuals, levels of uGSH were not affected by age in stroke patients (see Supplementary Figure S3).

In ischemic stroke patients, the influence of age on plasma sulfur-containing amino acid metabolites was also changed in a metabolite specific manner. For example, pHcy (R^2^ = 0.041, *P* = 0.004) and pCys (R^2^ = 0.124, *P* = 0.000) were positively correlated with age in ischemic stroke patients but not in healthy individuals (see Supplementary Figure S4). While pCysGly (R^2^ = 0.0487, *P* = 0.000) and pGSH (R^2^ = 0.0585, *P* = 0.000) significantly decreased with age in healthy individuals, these metabolites increased with age in ischemic stroke patients (pCysGly: R^2^ = 0.016, *P* = 0.073; pGSH: R^2^ = 0.040, *P* = 0.005) (see Supplementary Figure S4).

#### GFR

Relationships between GFR and urinary sulfur-containing amino acid metabolites were also different in ischemic stroke patients compared to healthy individuals. Specifically, in healthy individuals, GFR was significantly associated with uHcy (R^2^ = 0.037, *P* = 0.001), uCys (R^2^ = 0.055, *P* = 0.000), and uCysGly (R^2^ = 0.047, *P* = 0.000) (see Supplementary Figure S5). In ischemic stroke patients, these associations were attenuated (uHcy: R^2^ = 0.0099, *P* = 0.075; uCys: R^2^ = 0.0114, *P* = 0.075) or absent (uCysGly: R^2^ = 0.0002, *P* = ns) (see Supplementary Figure S5). However, GFR did not significantly influence uHTL and uGSH in healthy controls and ischemic stroke patients (see Supplementary Figure S5; Table 3).

**Table 3:** *P* values for correlations between urinary sulfur-containing amino acid metabolites and age, sex, GFR, in ischemic stroke patients and healthy individuals.

Variable	Other			Sulfur-containing amino acid metabolites				
	Age	Sex	GFR	uHcy	uCys	uCysGly	uGSH	uHTL
Healthy individuals, *P* values*								
Age				0.000(**−**)	0.000(**−**)	**0.000(−)**	ns	**0.021(−)**
Sex	0.001(**−**)			**0.000( +)**	**0.000( +)**	**0.000( +)**	ns	**0.009( +)**
GFR	0.000(**−**)	**0.022( +)**		**0.001( +)**	**0.000( +)**	**0.000( +)**	ns	ns
uCreatinine	0.000(**−**)	0.000( +)	**0.000( +)**	0.000( +)	0.000( +)	0.000( +)	0.000( +)	**0.000( +)**
Stroke patients, *P* values*								
Age				0.002(**−**)	0.005(**−**)	**ns**	ns	**ns**
Sex	0.000(**−**)			**ns**	**ns**	**ns**	ns	**ns**
GFR	0.000(**−**)	**ns**		**ns**	**ns**	**ns**	ns	ns
uCreatinine	0.000(**−**)	0.004( +)	**ns**	0.000( +)	0.000( +)	0.000( +)	0.000( +)	**ns**

*P values for associations affected by stroke are highlighted in bold. Signs (–) and ( +) show negative and positive correlations, respectively. GFR, glomerular filtration rate; ns, non-significant.

### Determinants of fibrin CLT and Abs_max_ in ischemic stroke patients and healthy individuals

#### Bivariate correlations

In healthy individuals, bivariate correlation analysis showed that fibrin CLT was associated with uCys, uCysGly, and ten other variables: *CBS T833C 844ins68* polymotphism (β = 0.12, *P* = 0.035), uCreatinine (β = − 0.18, *P* = 0.001), total cholesterol (β = 0.17, *P* = 0.003), LDL-cholesterol (β = 0.14, *P* = 0.022), triglycerides (β = 0.11, *P* = 0.062), BMI (β = 0.19, *P* = 0.001), hypertension (β = 0.17, *P* = 0.003), earlier CVD (β = 0.12, *P* = 0.043), age (β = 0.23, *P* = 0.000), and Abs_max_ (β = 0.55, *P* = 0.000) (Table 4).

**Table 4:** Determinants of plasma fibrin clot lysis time (CLT #36) and maximal absorbance (Abs_max_) in ischemic stroke patients and healthy controls.

	Ischemic stroke patients (n = 200)									Healthy controls (n = 291)							
Variable	**CLT**				**Abs**_**max**_					**CLT**				**Abs**_**max**_			
	Pearson correlation		Multiple regression		Pearson correlation		Multiple regression			Pearson correlation		Multiple regression		Pearson correlation		Multiple regression	
	β	*P*	β	*P*	β	*P*	β		*P*	β	*P*	β	*P*	β	*P*	β	*P*
uHTL	0.12	**0.110**	0.13	**0.048**	**−** 0.02	0.800				**−** 0.06	0.304			**−** 0.14	**0.018**	**−** 0.08 **−** 0.10	0.108 **0.028**^**$**^
uHcy	**−** 0.01	0.916			0.01	0.848				**−** 0.11	**0.054**			**−** 0.13	**0.043**		
uCys	0.04	0.592			**−** 0.07	0.337				**−** 0.15	**0.012**			**−** 0.14	**0.014**		
uCysGly	0.13	**0.079**	0.20	**0.003**	**−** 0.08	0.277				**−** 0.13	**0.023**			**−** 0.12	**0.038**		
uGSH	**−** 0.02	0.763			**−** 0.02	0.801				**−** 0.01	0.848			**−** 0.11	0.055	**−** 0.10 **−** 0.11	0.058 **0.018**^#^
uCreatinine	0.01	0.887			**−** 0.03	0.653				**−** 0.18	**0.002**			**−** 0.14	**0.014**		
pCysGly	**−** 0.14	**0.057**	**−** 0.22	**0.001**	0.11	0.134	0.17		**0.017**	0.01	0.846			0.00	0.951		
pGSH	**−** 0.17	**0.021**			0.01	0.933				0.10	0.101	0.11	**0.019**	**−** 0.04	0.544		
pMet	**−** 0.17	**0.021**			0.19	**0.009**	**−** 0.18		**0.009**	0.08	0.189			0.07	0.241		
*MTHFR C1298A*	0.15	**0.033**			0.21	**0.004**	0.18		**0.009**	**−** 0.03	0.610			0.06	0.305		
*MTHFR C677T*	**−** 0.02	0.767			0.02	0.819				0.06	0.281			0.05	0.392		
*CBS T833C 844ins68*	**−** 0.02	0.807			0.13	0.082	0.13		**0.049**	0.13	**0.024**	0.13	**0.008**	0.02	0.794		
*DHFR 19bpdel*	**−** 0.09	0.233			0.03	0.640				0.10	0.078			0.03	0.595		
Anti-*N*-Hcy	**−** 0.05	0.531			0.09	0.225				0.06	0.286			0.13	**0.028**		
Glucose	0.16	**0.031**			**−** 0.06	0.459				0.04	0.472			0.11	0.067		
Total cholesterol	**−** 0.13	0.076			**−** 0.07	0.355				0.17	**0.003**			0.22	**0.000**		
LDL cholesterol	**−** 0.07	0.311			**−** 0.07	0.376				0.14	**0.022**			0.22	**0.000**		
										0.04	0.504			**−** 0.07	0.241		
Triglycerides	0.00	0.962			**−** 0.14	0.063				0.11	**0.062**			0.13	**0.024**		
GFR	0.09	0.209	0.19	**0.007**	**−** 0.01	0.863				**−** 0.07	0.206			**−** 0.07	0.233		
BMI		NA				NA				0.19	**0.001**			0.29	**0.000**	0.11	**0.034**
Earlier CVD	0.16	**0.031**			0.00	0.971				0.12	**0.043**	0.13	**0.009**	0.03	0.610		
Hypertension	0.12	0.091			0.01	0.845				**0.17**	**0.004**			0.13	**0.024**		
Diabetes	0.13	0.080	0.19	**0.004**	**−** 0.11	0.134	**−** 0.11		0.131	**−** 0.04	0.534			**−** 0.02	0.692		
Other heart disease	**−** 0.02	0.834			**−** 0.05	0.530				0.10	0.085			**0.14**	**0.019**		
Age	0.09	0.259	0.10	0.139	0.04	0.537	0.05		0.460	0.23	**0.000**	0.02	0.704	0.33	**0.000**	0.20	**0.000**
Sex	**−** 0.23	**0.001**	**−** 0.21	**0.001**	**−** 0.01	0.843	0.08		0.240	**−** 0.08	0.187	**−** 0.12	**0.018**	0.05	0.359	0.12	**0.015**
Fibrin Abs_max_	0.29	**0.000**	0.35	**0.000**						0.55	**0.000**	0.55	**0.000**				
Fibrin CLT					0.29	**0.000**	0.33		**0.000**					0.55	**0.000**	0.48	**0.000**
			F = 8.5; *P* = 0.000, Adjust R^2^ = 0.24					F = 6.3, *P* = 0.000, Adjust R^2^ = 0.18				F = 26.1, *P* = 0.000, Adjust R^2^ = 0.34				F = 29.1; *P* = 0.000; Adjust R^2^ = 0.37	

*Variables included in analyses are shown by numerical entries. Empty fields show variables that were non-significant and were not included in a model.

^**$**^Model w/o uGSH. ^#^ Model w/o HTL. NA, not available; GFR, glomerular filtration rate; BMI, body mass index; CVD, cardiovascular disease; LDL, low-density lipoprotein; Abs_max_, maximum absorbance at 335 nm; CLT, clot lysis time; Anti *N*-Hcy, anti-*N*-Hcy-protein autoantibody. Hcy, homocysteine; HTL, Hcy-thiolactone; Cys, cysteine; CysGly, cysteinylglycine; Met, methionine. Urinary and plasma metabolites are shown by a letter ‘u’ or ‘p’, respectively, preceding the metabolite’s name.

In ischemic stroke patients, fibrin CLT was associated with a different set of variables that included pGSH (β = − 0.17, *P* = 0.021), pMet (β = − 0.17, *P* = 0.021), and five other variables: *MTHFR C1298A* polymorphism (β = 0.15, *P* = 0.033), glucose (β = 0.16, *P* = 0.031), sex (β = − 0.23, *P* = 0.001), earlier CVD (β = 0.16, *P* = 0.031), and Abs_max_ (β = 0.29, *P* = 0.000) (Table 4).

Fibrin Abs_max_ was associated with different sets of variables. In healthy individuals, fibrin Abs_max_ was associated with uHTL (β = − 0.14, *P* = 0.018), uHcy (β = − 0.13, *P* = 0.043), uCys (β = − 0.14, *P* = 0.014), uCysGly (β = − 0.12, *P* = 0.038), and ten other variables: uCreatinine (β = − 0.14, *P* = 0.014), anti-*N*-Hcy-protein autoantibody (β = 0.13, *P* = 0.028), total cholesterol (β = 0.22, *P* = 0.000), LDL-cholesterol (β = 0.22, *P* = 0.000), triglycerides (β = 0.13, *P* = 0.024), BMI (β = 0.29, *P* = 0.000), hypertension (β = 0.13, *P* = 0.024), other heart disease (β = 0.14, *P* = 0.019), age (β = 0.33, *P* = 0.000), and fibrin CLT (β = 0.55, *P* = 0.000) (Table 4). In stroke patients, fibrin Abs_max_ was associated with a different set of variables that included pMet (β = 0.19, *P* = 0.009) and three other variables: *MTHFR A1298C* polymorphism (β = 0.21, *P* = 0.004) and fibrin CLT (β = 0.29, *P* = 0.000) (Table 4).

#### Multiple regression analysis

In healthy individuals, multiple regression analysis showed that fibrin CLT was positively associated with pGSH (β = 0.11, *P* = 0.019) and four other variables: *CBS T833C 844ins68* polymorphism (β = 0.13, *P* = 0.008), earlier CVD (β = 0.13, *P* = 0.009), sex (β = − 0.12, *P* = 0.018), and fibrin Abs_max_ (β = 0.55, *P* = 0.000); adjusted R^2^ was 0.34, *P* = 0.000 (Table 4).

In ischemic stroke patients, fibrin CLT was associated with a different set of variables that included uHTL (β = 0.13, *P* = 0.048), uCysGly (β = 0.20, *P* = 0.003), pCysGly (β = − 0.22, *P* = 0.001) and four other variables: GFR (β = 0.19, *P* = 0.007), diabetes (β = 0.19, *P* = 0.004), sex (β = − 0.21, *P* = 0.001), and fibrin Abs_max_ (β = 0.35, *P* = 0.000); adjusted R^2^ was 0.24, *P* = 0.000 (Table 4).

In healthy individuals, fibrin Abs_max_ was associated with a different set of variables: uHTL (β = − 0.10, *P* = 0.025), uGSH (β = − 0.12, *P* = 0.012), and four other variables: BMI (β = 0.13, *P* = 0.013), age (β = 0.19, *P* = 0.000), sex (β = 0.12, *P* = 0.032), and fibrin CLT (β = 0.48, *P* = 0.000); adjusted R^2^ was 0.38, *P* = 0.000 (Table 4).

In stroke patients, fibrin Abs_max_ was associated with a different set of variables that included pCysGly (β = 0.17, *P* = 0.017), pMet (β = − 0.18, *P* = 0.009), and three other variables: *MTHFR A1298C* (β = 0.18, *P* = 0.009) and *CBS T833C 844ins68* (β = 0.13, *P* = 0.049) polymorphisms, and fibrin CLT (β = 0.31, *P* = 0.000); adjusted R^2^ was 0.18, *P* = 0.000 (Table 4).

These findings show that different sets of sulfur-containing amino acid metabolites and other variables contribute to fibrin clot properties in healthy individuals and stroke patients.

### Associations of sulfur-containing amino acid metabolites, fibrin CLT and Abs_max_ with ischemic stroke

#### Bivariate correlations

In bivariate correlation analysis, urinary (uHTL, uHcy, uCys, uGSH) and plasma (pHcy, pCys, pCysGly, pGSH) sulfur-containing amino acid metabolites, but not uCysGly, were significantly associated with ischemic stroke (Table 5) as were fibrin CLT and Abs_max_. Other variables such as pCreatinine, glucose, lipid measures, GFR, age, sex, and prior CAD, MI, hypertension, diabetes, and other heart diseases were also associated with ischemic stroke (Table 5).

**Table 5:** Determinants of ischemic stroke. Significant values are in bold.

Variable (n = 491; stroke n = 200, controls n = 291)	Bivariate correlations		Logistic regression					
			Model 1*		Model 2^**†**^		Model 3^**‡**^	
	β	*P*	B	*P*	B	*P*	B	*P*
uHTL	− 0.16	**0.000**	**−** 0.01	**0.010**	**−** 0.01	**0.008**	**−** 0.01	**0.007**
uHcy	0.19	**0.000**	0.10	**0.035**	0.12	**0.031**	**0.10**	0.078
uCys	0.19	**0.000**	0.01	**0.027**	0.01	**0.045**	0.01	**0.039**
uCysGly	0.06	0.189		ns		ns		ns
uGSH	**−** 0.23	**0.000**	**−** 0.11	**0.003**	**−** 0.31	**0.025**	**−** 0.13	**0.005**
uCreatinine	**−** 0.07	0.127		ns		ns		ns
pHcy	0.24	**0.000**		**ns**		**ns**		**ns**
pCys	0.26	**0.000**	0.01	**0.000**	0.01	**0.001**	**0.01**	**0.003**
pCysGly	**−** 0.11	**0.016**	**−** 0.08	**0.005**	**−** 0.08	**0.011**	**−** **0.07**	**0.035**
pGSH	**−** 0.16	**0.000**		ns		ns		ns
pCreatinine	0.32	**0.000**	0.04	**0.000**	0.04	**0.001**		ns
Age	0.53	**0.000**	0.06	**0.000**	0.05	**0.001**	0.05	**0.003**
Sex	0.17	**0.000**		ns		ns		ns
Anti-*N*-Hcy	0.14	**0.002**		ns		ns		ns
GFR	**−** 0.45	**0.000**						ns
Glucose	0.24	**0.000**						ns
LDL cholesterol	**−** 0.18	**0.000**						ns
HDL cholesterol	**−** 0.28	**0.000**						ns
Triglycerides	0.11	**0.008**						ns
Hypertension	0.52	**0.000**			1.28	**0.000**	1.20	**0.001**
Other heart disease	0.28	**0.000**			1.23	**0.024**	1.13	**0.046**
Early CAD	0.48	**0.000**				ns		ns
Early MI	0.20	**0.000**				ns		ns
Diabetes	0.31	**0.000**				ns		ns
*MTHFR C677T*	0.07	0.088	0.54	**0.037**	**−** 0.67	**0.023**	**−** 0.69	**0.029**
*MTHFR A1298C*	0.05	0.282		ns		ns		ns
*CBS T833C 844ins68*	**−** 0.06	0.135		ns		ns		ns
Fibrin CLT	0.16	**0.001**		ns		ns		ns
Fibrin Abs_max_	0.21	**0.000**	7.1	**0.049**	10.6	**0.007**	10.9	**0.010**
* Variables included in each model are shown by numerical or textual entries. Ischemic stroke was coded as 1, no stroke as 0			**−** 2 log likelihood = 311.8, Cox & Snell R^2^ = 0.48, Nagelkerke R^2^ = 0.64; % Correct 84.5		**−** 2 log likelihood = 266.9, Cox & Snell R^2^ = 0.52, Nagelkerke R^2^ = 0.71; % Correct 87.6		**−** 2 log likelihood = 250.8, Cox & Snell R^2^ = 0.53, Nagelkerke R^2^ = 0.71; % Correct 87.6	

GFR, glomerular filtration rate; CAD coronary artery disease; MI, myocardial infarction; CLT, clot lysis time; LDL, low-density lipoprotein; Anti *N*-Hcy, anti-*N*-Hcy-protein autoantibody. Hcy, homocysteine; HTL, Hcy-thiolactone; Cys, cysteine; CysGly, cysteinylglycine; Abs_max_, maximum absorbance at 335 nm; CLT, clot lysis time; ns, non-significant. Urinary and plasma metabolites are shown by a letter ‘u’ or ‘p’, respectively, preceding the metabolite’s name.

#### Logistic regression

In logistic regression analysis, adjusting for anti-*N*-Hcy autoantibodies^14^, age, and sex, uHTL, uHcy, uCys, uGSH, pCys, and pCysGly remained significantly associated with ischemic stroke while pHcy and pGSH did not (Model 1, Table 5). *MTHFR C677T* polymorphism that affects the MTHFR enzyme, important for B-vitamin-dependent recycling of Hcy to methionine^15^, was also associated with ischemic stroke in Model 1 (Table 5), consistent with earlier studies^16^.

Adjustments for earlier diseases did not affect these associations (Model 2, Table 5). Other adjustments for GFR, glucose, LDL cholesterol, HDL cholesterol, and triglycerides, also did not affect these associations (Model 3, Table 5), except for uHcy which was not significantly associated with stroke in Model 3. The associations of sulfur-containing amino acid metabolites and *MTHFR C677T* polymorphism with ischemic stroke were independent of other metabolites and traditional stroke risk factors such as lipid measures, GFR, glucose, age, sex, early CAD, MI, hypertension, diabetes, and other heart diseases.

Logistic regression analysis in a model adjusted for anti-*N*-Hcy autoantibodies^14^, age, and sex, also showed that fibrin Abs_max_, but not CLT, was significantly associated with ischemic stroke (*P* = 0.049, Model 1, Table 5). The association of fibrin Abs_max_, with stroke became stronger in models adjusted for earlier diseases (*P* = 0.007, Model 2, Table 5) and for GFR, glucose, LDL cholesterol, HDL cholesterol, and triglycerides (Model 3, Table 5). These findings show that the association of fibrin Abs_max_ with ischemic stroke was independent of sulfur-containing amino acid metabolites and traditional stroke risk factors such as lipid measures, glucose, GFR, age, sex, and earlier CAD, MI, hypertension, diabetes, and other heart diseases (Table 5).

### Contribution of individual sulfur-containing amino acid metabolites to the risk of ischemic stroke

To estimate the contribution of individual sulfur-containing amino acid metabolites to the risk of ischemic stroke, we examined effects of each of these metabolites on R^2^ values in the logistic regression models described in Table 5. We found that uHTL, uHcy, uCys, and uGSH explained individually 1, 0.5, 0.5, and 1.5%, respectively, of the ischemic stroke risk, with all four urinary metabolites explaining up to 6.5% of the risk (Model 3, Table S4). pCys and pCysGly individually explained 1.5 and 0.5%, respectively, of the ischemic stroke risk, with all four plasma metabolites explaining 2% of the risk. Notably, pHcy and pGSH did not contribute to the risk of stroke. Similar values were obtained using Model 1 (Table S4). Urinary sulfur-containing amino acid metabolites explained 6.5–9% stroke risk compared to just 2–2.5% explained by the plasma metabolites. Urinary and plasma sulfur-containing amino acid metabolites together explained 9–14% to the ischemic stroke risk, with the greatest contribution from pCys (1.5–2%) and uGSH (1–1.5%) (Table S4).

## Discussion

We found that (i) sulfur-containing amino acid metabolites such as uHTL, uHcy, uCys, uGSH, pCys, and pCysGly were independently associated with stroke; (ii) fibrin Abs_max_ was associated with stroke; (iii) uHTL and uGSH were associated with fibrin Abs_max_ while pGSH was associated with fibrin CLT in healthy individuals. (iv) pCysGly, pGSH, and pMet were associated with fibrin Abs_max_ while uHTL, uCysGly and pCysGly were associated with fibrin CLT in stroke patients.

In addition to stroke-associated changes in levels of sulfur-containing amino acid metabolites, we found profound changes in the relationships of these metabolites to fibrin clot properties, age, and GFR in stroke patients. For example, negative associations of fibrin clot properties (CLT, Abs_max_) with uCys (Fig. 3A,B) and uHTL (Fig. 3C) observed in healthy individuals were not seen in stroke patients (Fig. 3D–F). Further, negative associations of fibrin clot properties (CLT, Abs_max_) with uHcy, uCysGly, and uGSH in healthy individuals were not seen in stroke patients (Figure S2). Negative associations between urinary sulfur-containing amino acid metabolites (uHTL, uCysGly) and age in healthy individuals were also not seen in stroke patients (see Supplementary Figure S3). Moreover, negative associations between plasma sulfur-containing amino acid metabolites (pCysGly, pGSH) and age in healthy individuals were either not seen in stroke patients (pCysGly) or were changed to positive association (pGSH) (see Supplementary Figure S3). Two other plasma sulfur-containing amino acid metabolites (pHcy, pCys) were positively associated with age in stroke patients but were not affected by age in healthy individuals (see Supplementary Figure S4). Positive associations between urinary sulfur-containing amino acid metabolites (uHcy, uCys, uCysGly) and GFR in healthy individuals were not seen in stroke patients (see Supplementary Figure S5). These changes suggest profound metabolic rewiring that affects sulfur amino acid metabolism associated with stroke. Whether these changes were causally related to stroke is still to be figured out in future studies.

Accumulating evidence suggests that impaired fibrin clot properties are associated with stroke^7,8^. In the present study we found that CLT and Abs_max_ were significantly correlated with each other in healthy individuals (β = 0.55, *P* = 0.000) and that this correlation was attenuated in stroke patients (β = 0.29, *P* = 0.000). Notably, we found that the CLT *vs*. Abs_max_ correlation was sex-specific in stroke patients and was seen in women (Fig. 1D) but not in men (Fig. 1B). The size of this correlation in stroke patients was like that previously reported in CAD patients (β = 0.23, *P* = 0.000)^13^. Other studies reported similar correlations between CLT and Abs_max_ in patients with diabetes^17,18^ and in healthy individuals^9^.

In the present study we showed that sulfur-containing amino acid metabolites were associated with fibrin clot properties in a disease status-dependent manner. Specifically, in healthy individuals uHTL and uGSH were associated with Abs_max_ while pGSH was associated with CLT (Table 4). A different set of metabolites was associated with fibrin clot properties in stroke patients: pCysGly and pMet were associated fibrin Abs_max_ while uHTL, uCysGly, and pCysGly were associated fibrin CLT. These associations have not been reported before.

Our present finding that uHTL was associated with fibrin CLT in ischemic stroke patients together with an earlier finding that uHTL was associated with CLT in CAD patients^13^ suggests that uHTL can affect fibrin clot properties in heart and brain diseases. These findings in conjunction with the present finding that uHTL was associated with Abs_max_ but not CLT in healthy individuals suggest that associations of uHTL with specific fibrin clot properties such as CLT and Abs_max_ can be disease status specific. This suggestion is supported by previous findings showing that other metabolites such as HDL cholesterol, triglycerides, pCreatinine (which predicted Abs_max_ in CAD patients^13^), and other variables such as GFR (which predicted CLT in CAD patients^13^), were not associated with Abs_max_ nor CLT in stroke patients in the present study (Table 4).

The disparate influences of uHTL (in stroke patients and healthy controls), uGSH and pGSH (in healthy controls), pCysGly (in stroke patients), uCysGly and pMet (in stroke patients) on CLT and Abs_max_ (Table 4) suggest that each metabolite can affect fibrin clot properties via metabolite-specific mechanisms. For example, modification of protein lysine residues by HTL produces *N*-Hcy-protein in a process called *N*-homocysteinylation, which results in protein damage^11^ and generation anti-*N*-Hcy-protein antibodies^14^. Under normal circumstances in healthy individuals, HTL is efficiently cleared by excretion into urine by the kidney^19^. However, in stroke patients, kidney function is impaired as shown by significantly reduced GFR and uCreatinine, and significantly elevated pCreatinine (see Supplementary Table S2). Notably, we found that uHTL levels were lower in stroke patients and were accompanied by higher plasma levels of anti-*N*-Hcy-protein antibodies compared to healthy individuals (Table S2). These findings suggest that the increase in anti-*N*-Hcy-protein antibody levels in stroke patients is caused by HTL accumulation in the plasma due to its impaired urinary clearance (see Supplementary Table S2). The accumulation of HTL in the human body elevates *N*-Hcy-protein levels^20^, including *N*-Hcy-fibrinogen and *N*-Hcy-albumin^21^, which stimulate the generation of anti-*N*-Hcy-protein antibodies^14^. Apart from their ability to generate an autoimmune response^14^, *N*-Hcy-proteins that are present in the human plasma^22^ can be prothrombotic as demonstrated for *N*-Hcy-fibrinogen, which formed fibrin clots that were more resistant to lysis by plasmin^23^.

Alternatively, low-molecular weight plasma thiols such as pCysGly and pGSH can affect fibrin clot properties by binding via disulfide bonds to plasma proteins that become components of the fibrin clot. In fact, *S*-CysGly- and *S*-GSH-bound forms of albumin and globulin have been identified in human plasma with *S*-CysGly-globulin and *S*-CysGly-albumin representing 40–50% of the total thiolated proteins^24^. Although *S*-Cys-globulin and *S*-pCys-albumin levels are comparable to the levels of* S*-pCysGly-globulin and *S*-pCysGly-albumin, plasma Cys was not associated with fibrin clot properties, suggesting a specific influence of plasma CysGly and possibly *S*-CysGly-modified proteins on fibrin clot properties.

We found that sulfur-containing amino acid metabolites, uHTL, uGSH, and pCysGly, were associated both with fibrin clot properties (Table 4) and stroke (Table 5). Of these, uHTL has been previously shown to affect fibrin clot properties^13^ and predict MI in CAD patients^12^, suggesting that HTL can contribute to stroke and CAD via similar mechanisms involving protein modification by *N*-homocysteinylation^11^.

*MTHFR A1298C* and *MTHFR C677T* polymorphisms affect the MTHFR enzyme, important for B-vitamin-dependent recycling of Hcy to methionine^15^. In the present study, *MTHFR A1298C* polymorphism was associated with fibrin clot properties (Table 4) while *MTHFR C677T* polymorphism was associated with stroke (Table 5). The association of *MTHFR A1298C* polymorphism with fibrin clot properties has been reported before^25^. Although *MTHFR A1298C* polymorphism has been reported to be associated with stroke^26^, in the present study there was no association either in bivariate or logistic regression analyses (Table 5). However, in the present study we found that *MTHFR C677T* polymorphism was associated with stroke (Table 5), consistent with earlier studies^16^.

Of the sulfur-containing amino acid metabolites associated with stroke (uHTL, uHcy, uCys, uGSH, pCys, and pCysGly) (Table 5) some were also associated with fibrin clot properties (uHTL, uGSH, pCysGly) (Table 4). Other variables such as total cholesterol, LDL cholesterol, GFR, and age were also associated both with fibrin clot properties (Table 4) and stroke (Table 5). Taken together, these findings suggest that the sulfur-containing amino acid metabolites uHTL, uGSH, and pCysGly as well as the other variables can promote stroke by promoting unfavorable fibrin clot properties.

We also found factors that were not associated with fibrin clot properties but were nevertheless associated with stroke. Among those were the sulfur-containing amino acid metabolites uHcy and uCys. These findings suggest that uHcy and uCys can promote stroke without influencing fibrin clot properties.

In the present study we found that in logistic regression analyses, plasma and urinary sulfur-containing amino acid metabolites were associated with ischemic stroke, independently of established risk factors (Table 5). To our best knowledge, the independent associations of uHTL, uHcy, uCys, uGSH, pCys, and pCysGly with ischemic stroke have not been previously reported. The association of pHcy with stroke has been reported before^27–29^. Although in the present study pHcy was associated with stroke in unadjusted analyses, this association disappeared after adjustments for GFR, glucose, LDL cholesterol, HDL cholesterol, and earlier diseases (Table 5). Our findings suggest that sulfur-containing amino acid metabolites such as uHTL, uHcy, and pCysGly might be better predictors of stroke than pHcy. The role of sulfur-containing amino acids in stroke, and their diagnostic value remain to be investigated in future studies.

### Strength and limitations

The present study is the first to evaluate sulfur-containing amino acid metabolites as determinants of fibrin clot properties in relation to stroke events. The case–control design of the present study allows the identification of associations whose mechanistic significance and causality need to be assessed in prospective studies. As our study was limited to Caucasian populations, our findings are still to be confirmed in other populations.

### Conclusions

Sulfur-containing amino acid metabolites such as uHTL, uGSH, pCysGly were associated both with fibrin clot properties and stroke, suggesting that these metabolites can promote stroke by promoting unfavorable fibrin clot properties. Other sulfur-containing amino acid metabolites such as uHcy, uCys, pCys, and factors such as *MTHFR C677T* polymorphism were associated with stroke without influencing fibrin clot properties. Targeting sulfur-containing amino acid metabolites and their urinary excretion might be a useful therapeutic strategy to mitigate prothrombotic phenotypes that increase a risk of stroke.

## Materials and methods

### Patients

The present study included ischemic stroke patients (n = 200) and healthy control individuals (n = 291). Participant characteristics and blood/urine samples were collected at the admission to the study. Serum, citrated plasma, and EDTA plasma were prepared as previously described^13^. Stroke patients had earlier coronary stenosis (23.3%) (> 50% of cross-surface area obstructed in at least one of the major coronary arteries), myocardial infarction (8.7%), other heart disease (22.5%), diabetes (24.0%), and hypertension (77.5%). The frequency of these risk factors was up to 10 times less prevalent in control individuals: 2.3%, 0.7%, 4.3%, 3.7%, and 21.2%, respectively. Stroke patients were on medications at admission (78.3%), including antiplatelet drugs (11%), acetyl salicylic acid (30%), statins (30%), β-blockers (41%), and metformin (19%). Medication use in control individuals was 7.7%, including antihypertensives (3%), statins/anti-lipid (2%), β-blockers (1.4%), and metformin (0.3%). Samples were assayed by investigators blinded to the clinical data to avoid bias. Written informed consent was obtained from all the participants on the admission to the study. The study protocols were following the principles of the Declaration of Helsinki and was approved by the Bioethical Committee, Poznań University of Medical Sciences (#908/17, #909/17, approved Sep 2017; #1240/17, approved Dec 2017).

### The clotting/lysis assay

The assay was changed from that described previously^9^. Briefly, 25 µL citrated plasma was added to 75 µL buffer (50 mM Tris–HCl, 150 mM NaCl, pH 7.6) containing 12.5 ng of tPA (Molecular Innovations), 83 ng/ml final concentration. The reaction was started with 50 µL of activation mix containing 0.09 U/mL of thrombin (Millipore-Sigma) and 22.5 mM CaCl_2_ in 50 mM Tris–HCl, 150 mM NaCl (pH 7.6) to each well of the 96-well plate using a multichannel pipette at 20 s intervals. The time of addition of the activation mix was recorded to enable the plate reader times to be adjusted to the start of clot initiation. Absorbance was read at 340 nm every 30 s for 1 h in a Tecan NanoQuant Infinite M200 Pro microplate reader. Complete lysis of fibrin clots occurred within 1 h at the tPA concentration used. Plasma samples were assayed in duplicates and the values were averaged.

### Clotting/lysis data analysis

Kinetics of fibrin clot formation and lysis, illustrated in Supplemental Figure S1, were analyzed using a customized software kindly provided by Dr. Peter Grant^9^. Maximum absorbance at 340 nm (Abs_max_, a measure of fibrin network density) and fibrin CLT (*i.e.*, a time at which A_340_ was reduced to 50% of the highest value, a measure clot’s susceptibility to lysis) were calculated from the kinetics. Inter-assay variabilities for Abs_max_ and fibrin CLT were 1.5% and 4.7%, respectively. Correlations between the clotting and lysis variables for the stroke patients and the healthy individuals are shown in Supplementary Table S1.

### Genotyping

Polymorphisms were established by using the PCR-RLFP (*MTHFR C677T* rs1801133, *DHFR 19bpdel* rs70991108, and *CBS T833C* rs5742905/*CBS 844ins68*) or TaqMan probes (ThermoFisher Scientific) (*MTHFR A1298C* rs1801131) according to previously published methods^30,31^.

### Metabolite assays

Plasma and urinary sulfur-containing amino acids metabolites^32,33^ and uHTL^34^ were quantified as previously described. Creatinine and lipids were quantified by standard assays^13^.

### Anti-*N*-Hcy-protein autoantibody assays

Anti-*N*-Hcy-protein autoantibodies were quantified as previously described^14,35,36^.

### Statistics

Normality of distribution was tested with the Shapiro–Wilk’s statistic. Mean ± standard deviation (SD) or median was calculated for normally or non-normally distributed variables, respectively. An unpaired two-sided *t*-test was used for comparisons between two groups of variables with normal distribution. A Mann–Whitney rank sum test was used for comparisons between two groups of non-normally distributed variables. Associations between fibrin clot lysis time (CLT) or Abs_max_ and other variables were studied by bivariate correlations, multiple regression, and logistic regression analyses. Statistical software packages Statistica, version 13 (TIBCO Software Inc., Palo Alto, CA, USA) and PSPP, version 1.0.1 (www.gnu.org) were used. Probability values were 2-sided and *P* value < 0.05 was considered significant.

### Supplementary Information

Supplementary Information.

## Data Availability

The data that support the findings of this study are available in the methods and/or supplementary material of this article.
