# Supplementary material for: Homocysteine thiolactone and other sulfur-containing amino acid metabolites are associated with fibrin clot properties and the risk of ischemic stroke
Source: Sci Rep. 2024 May 16;14:11222. doi: 10.1038/s41598-024-60706-2 (PMC11099160; doi:10.1038/s41598-024-60706-2)
Supplement: Supplementary file 1 — Supplementary Information. [file 41598_2024_60706_MOESM1_ESM.docx]

**Homocysteine thiolactone and other sulfur-containing amino acid metabolites are associated with fibrin clot properties and the risk of ischemic stroke**

Marta Sikora ^1^, Ewa Bretes ^2^, Joanna Perła-Kaján ^2^, Olga Utyro ^2^, Kamila Borowczyk ^3^, Justyna Piechocka ^3^, Rafał Głowacki ^3^, Izabela Wojtasz ^4^, Radosław Kaźmierski ^5,6^ & Hieronim Jakubowski ^2, 7,^*

**Supplementary Information**

**Figure S1.** Illustration of clotting and lysis variables. Variables examined in the present study, fibrin CLT and fibrin Abs_max_, are highlighted in bold.

**Figure S2.** Stroke abrogates influence of urinary sulfur-containing amino acid metabolites on fibrin clot properties. Levels of uHcy (A-D), uCysGly (E-H), and uGSH (I, J) are plotted vs. fibrin CLT (A, C, E, G) and Abs_max_ (B, D, F, H) in healthy individuals (A, B, E, F, I) and ischemic stroke patients (C, D, G, H, J).

**Figure S3.** Relationships between age and urinary sulfur-containing amino acid metabolites. Levels of uHcy (A, B), uCys (C, D), uCysGly (E, F), uHTL (G, H), and uGSH (I, J) are plotted vs. age in healthy individuals (A, C, E, G, I) and ischemic stroke patients (B, D, F, H, J).

**Figure S4.** Relationships between age and plasma sulfur-containing amino acid metabolites. Levels of pHcy (A, B), pCys (C, D), pCysGly (E, F), and pGSH (G, H) are plotted vs. age in healthy individuals (A, C, E, G) and ischemic stroke patients (B, D, F, H).

**Figure S5.** Relationships between glomerular filtration rate (GFR) and urinary sulfur-containing amino acid metabolites. Levels of uHcy (A, B), uCys (C, D), uCysGly (E, F), uHTL (G, H), and uGSH (I, J) are plotted vs. GFR in healthy individuals (A, C, E, G, I) and ischemic stroke patients (B, D, F, H, J).

**Table S1.** Descriptive statistics of the variables analyzed in the present study.

**Table S2.** Pearson correlation coefficients for relationships between turbidimetric clotting and lysis variables in ischemic stroke patients and healthy individuals.

**Table S3**. *P* values for correlations between fibrin clot properties vs. plasma sulfur-containing amino acid metabolites and creatinine (pCreat) in ischemic stroke patients and healthy individuals.

**Table S4**. Contribution of urinary and plasma sulfur-containing amino acid metabolites to the risk of ischemic stroke.

**Figure S1.**


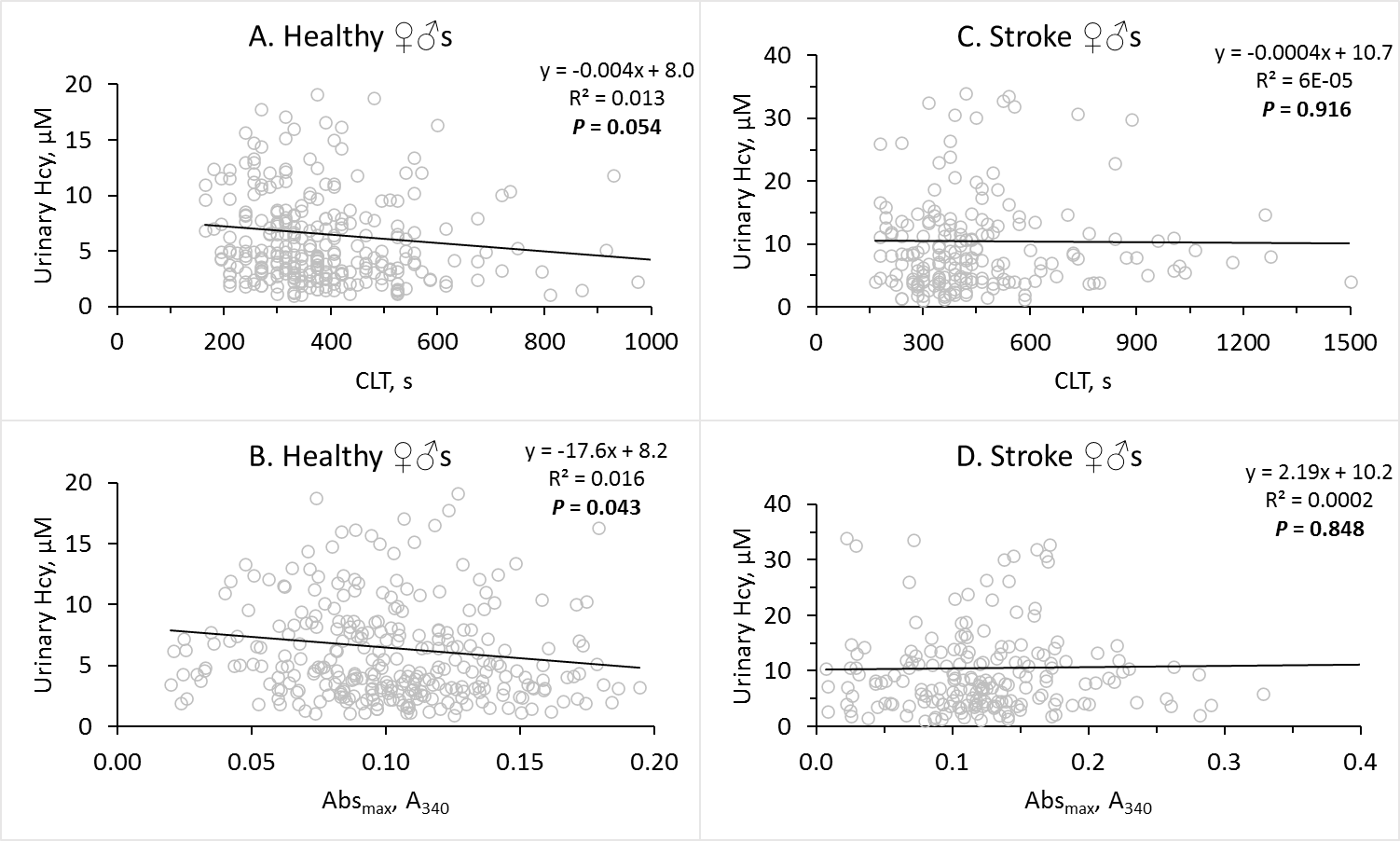

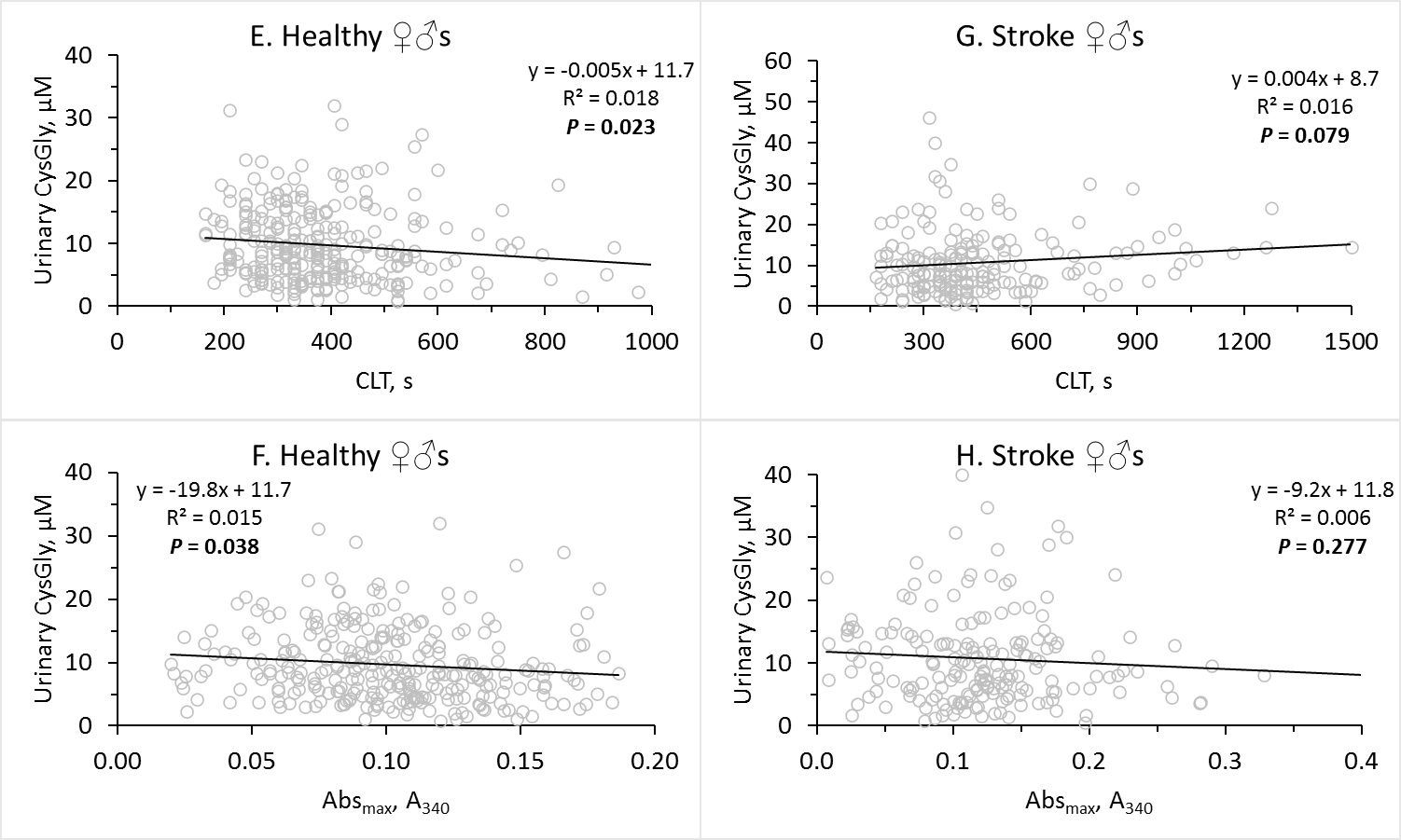

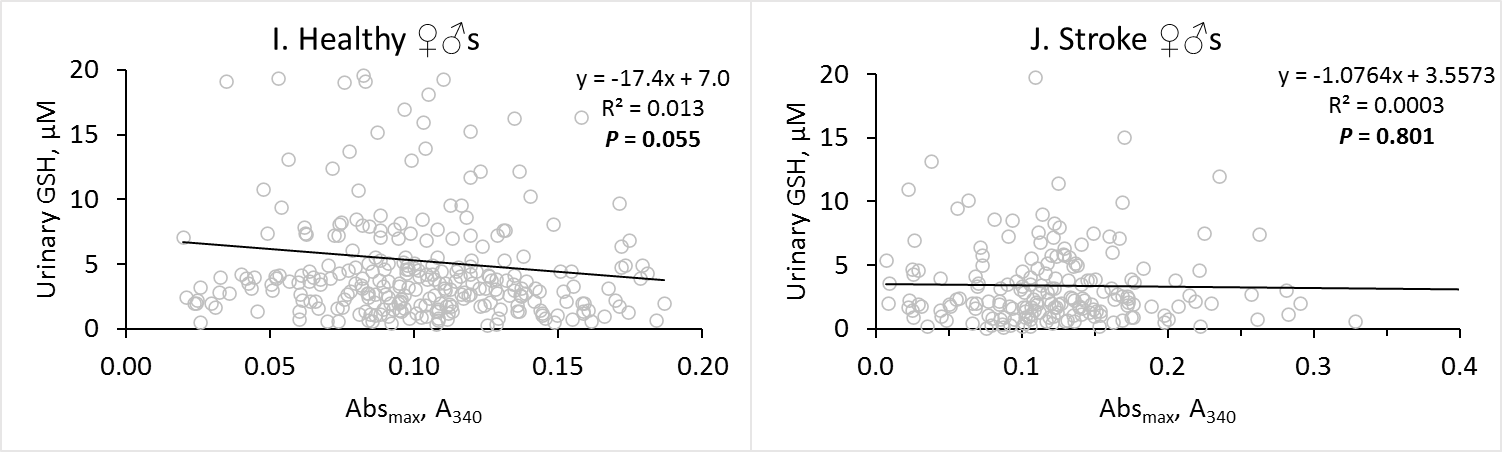


**Figure S2**


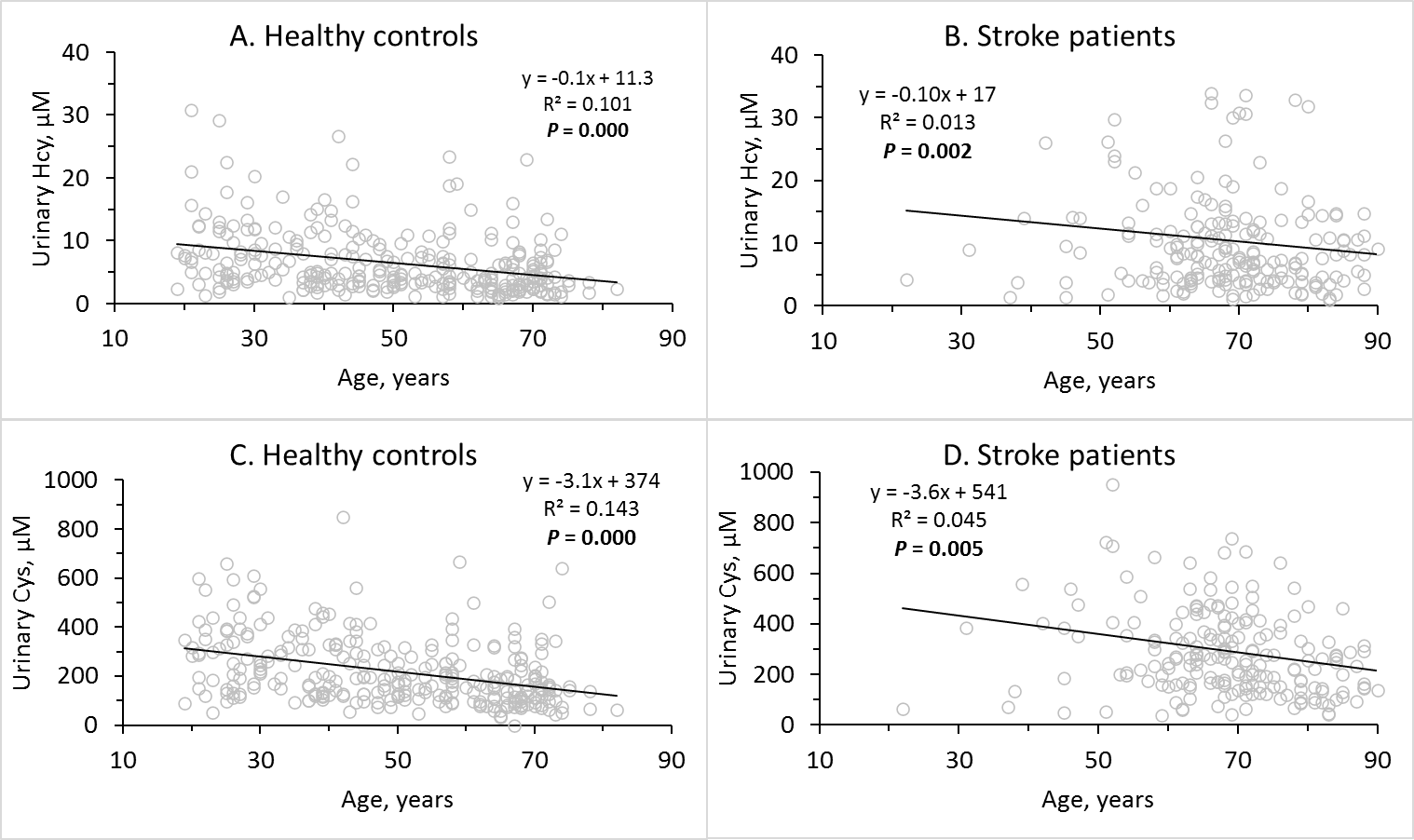

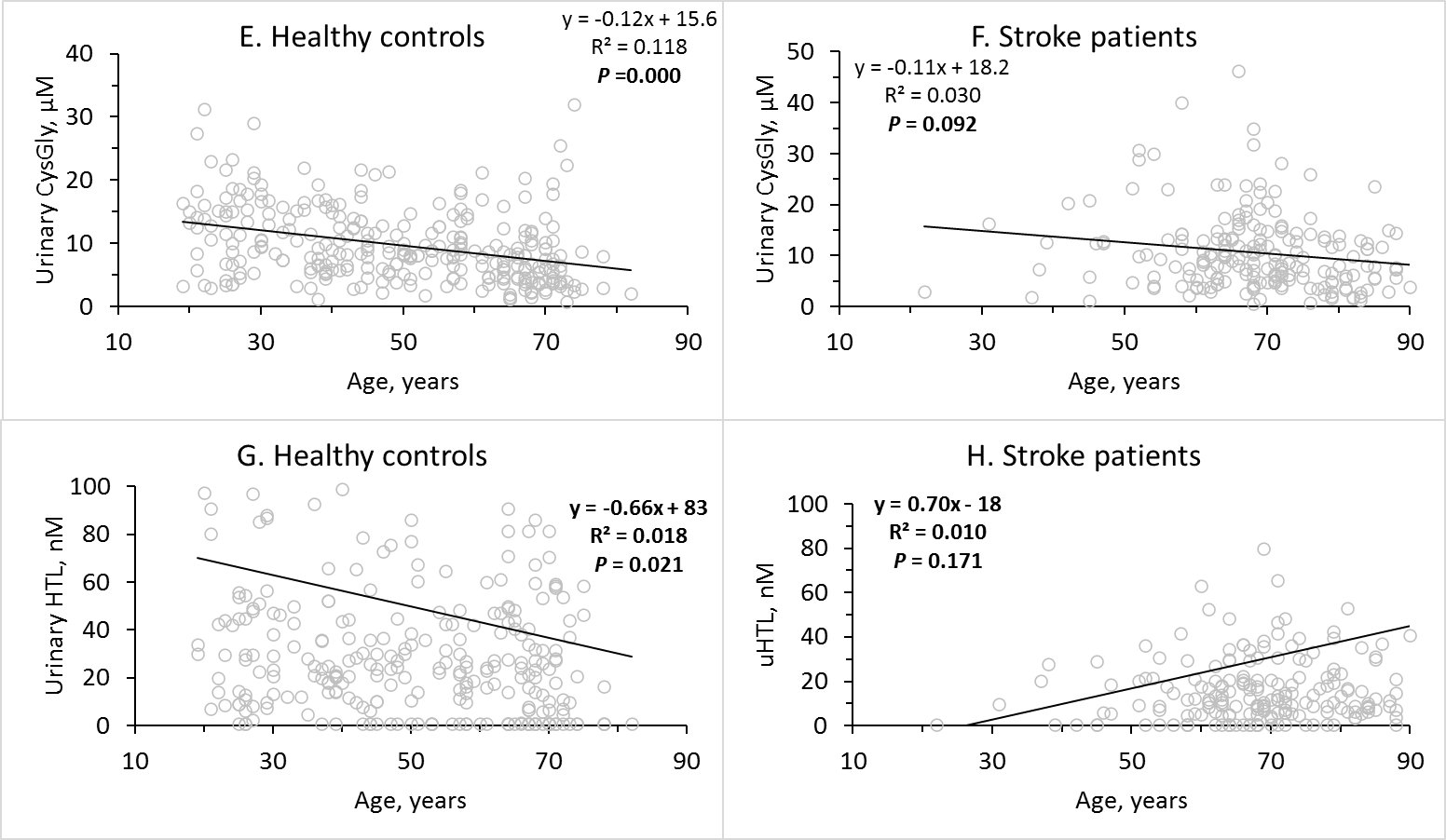


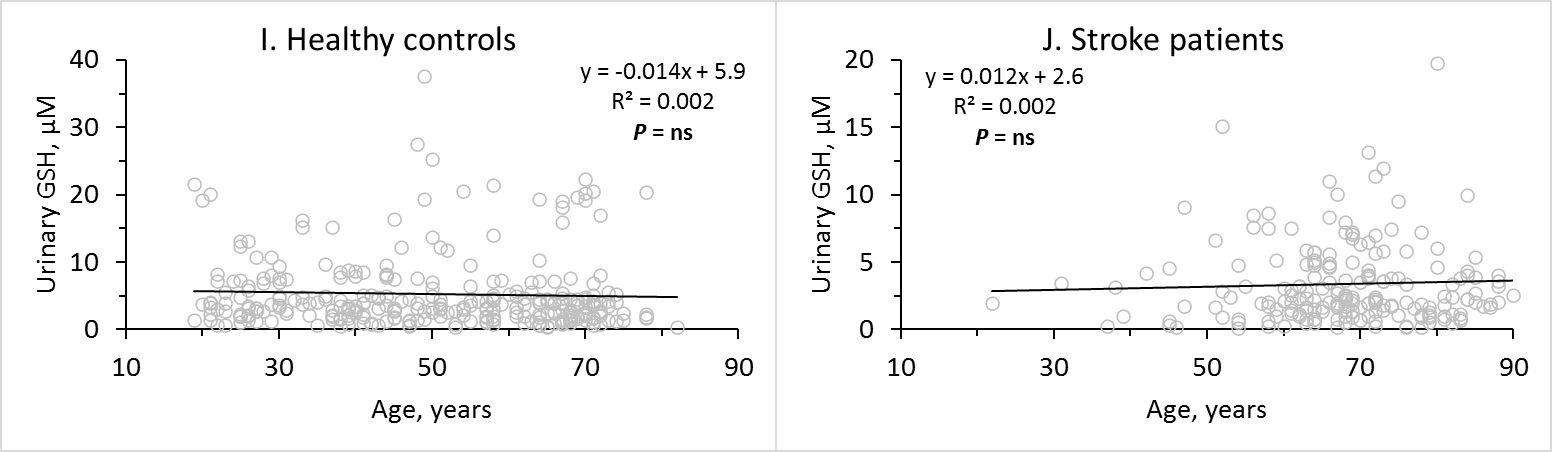


**Figure S3**


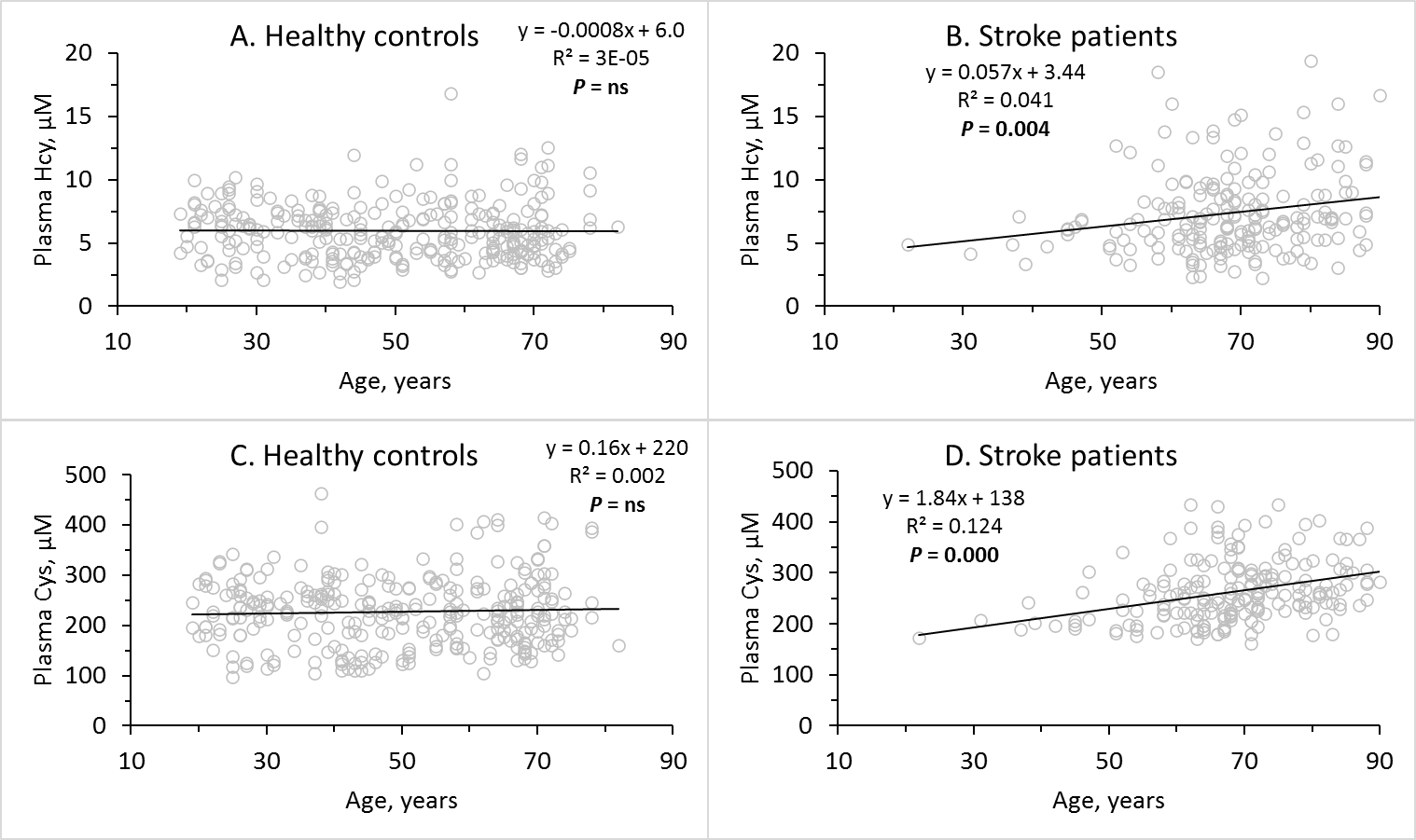

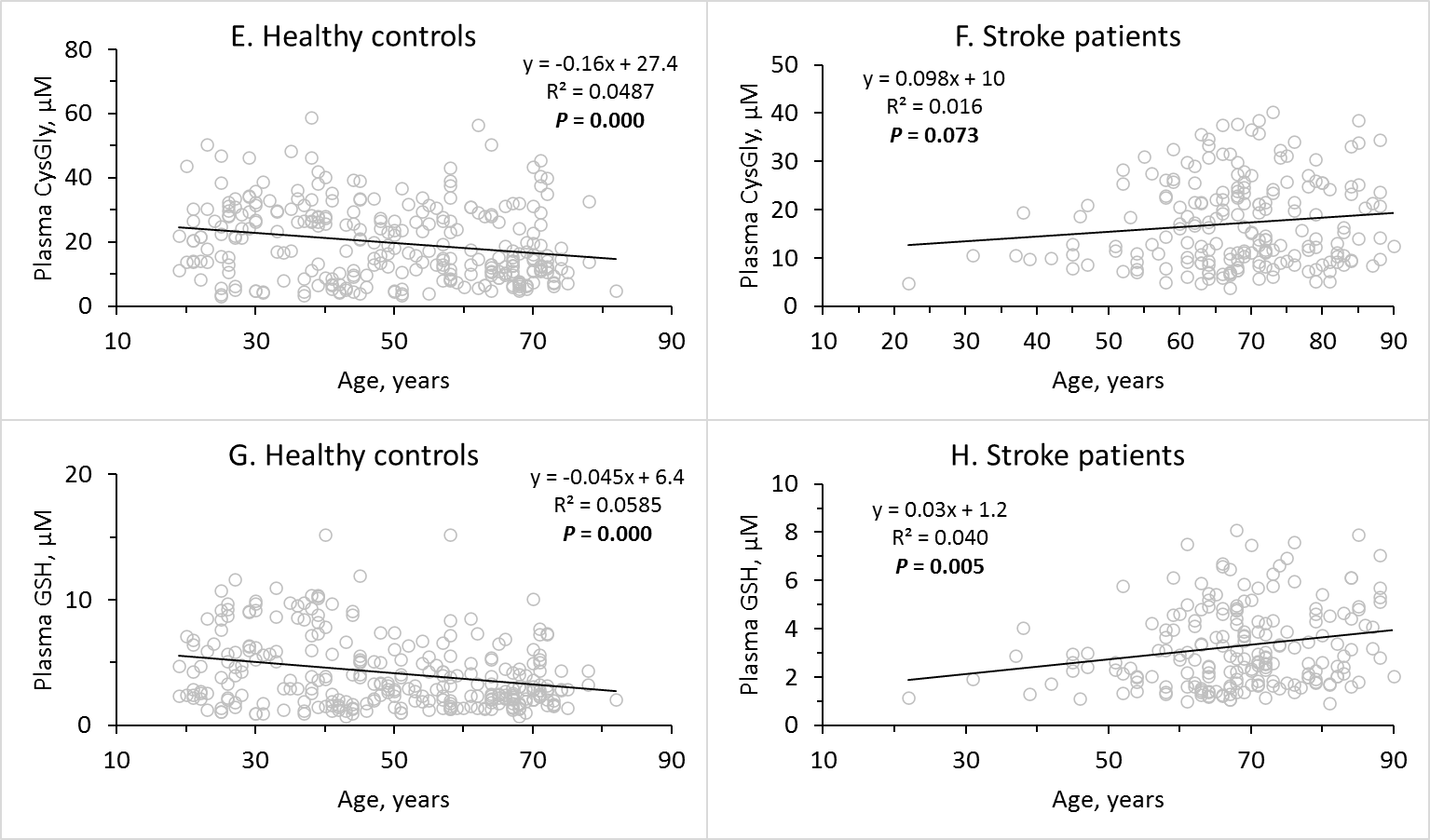


**Figure S4**

**
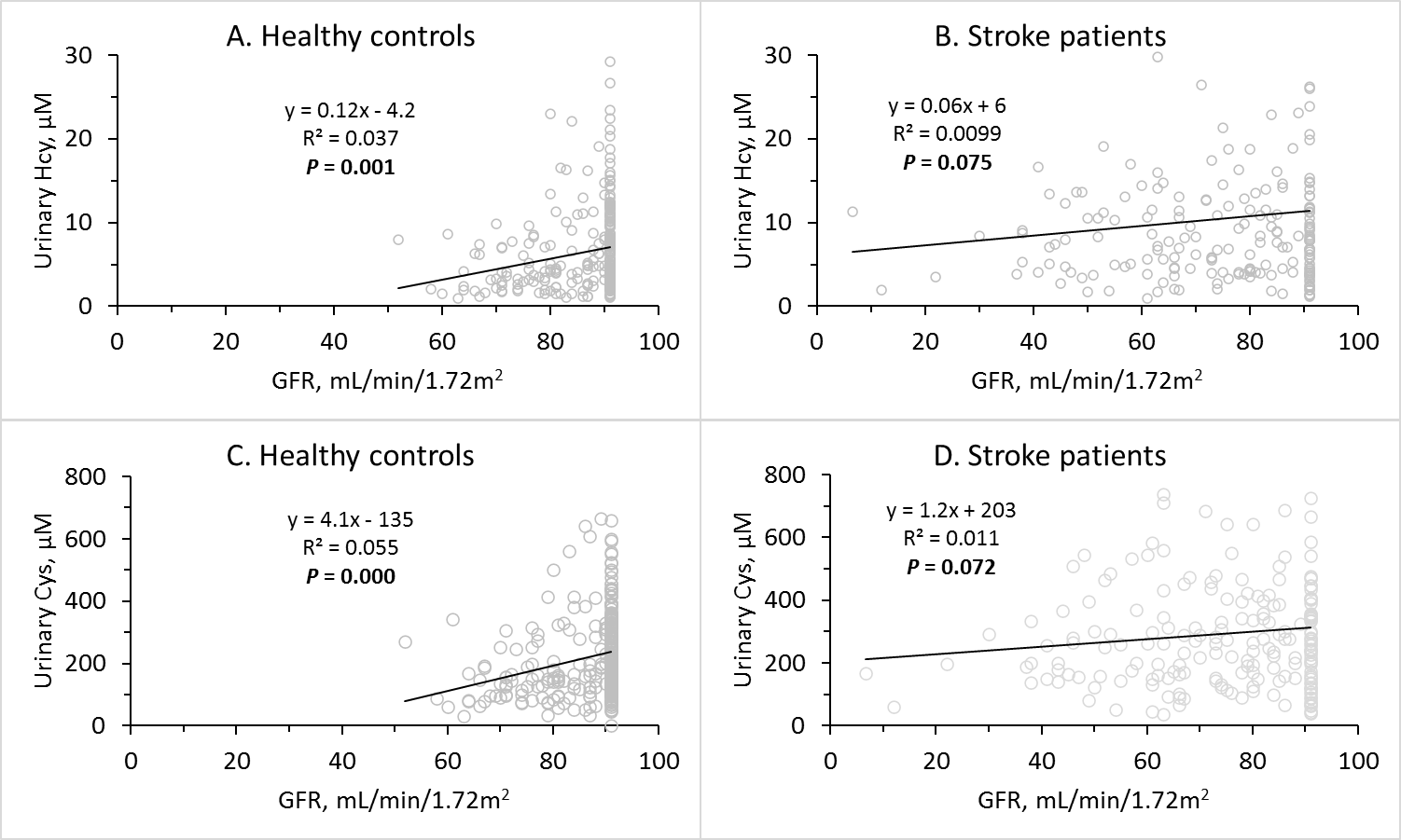
**

**
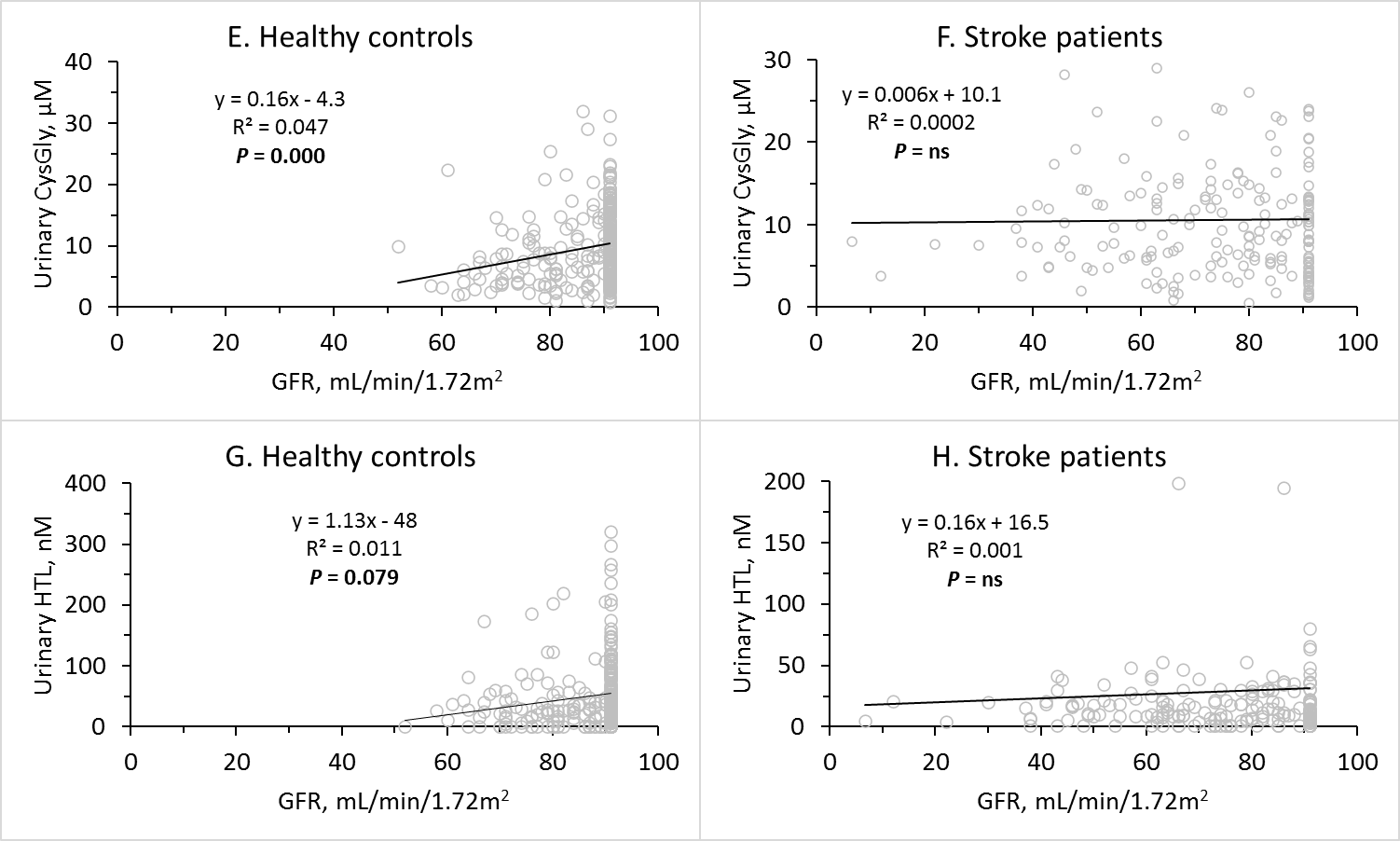
**

**
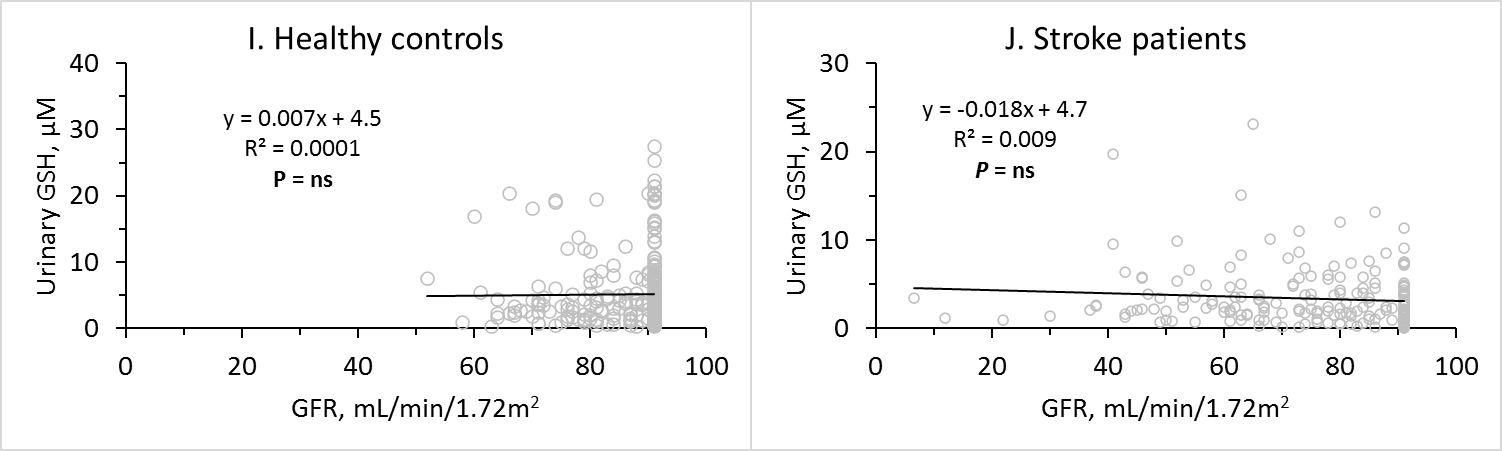
Figure S5**

| **Table S1. Characteristics of stroke patients and healthy controls*.** | | | |
| --- | --- | --- | --- |
| Variable | Stroke patients  (n = 191) | Healthy controls  (n = 291) | *P* value |
|  | | | |
| Fibrin clot properties | | | |
| Fibrin Abs_max_, A_340_ | 0.123±0.065 | 0.101±0.036 | 0.000 |
| Fibrin CLT, s | 452±226 | 391±154 | 0.001 |
| Urinary metabolites | | | |
| uHcy, μM | 10.4±10.2 | 6.5±5.0 | 0.000 |
| uCys, μM | 293±199 | 221±136 | 0.000 |
| uCysGly, μM | 10.6±7.5 | 9.7±5.8 | 0.136 |
| uGSH, μM | 3.4±3.3 | 5.3±5.4 | 0.000 |
| uHTL, nM | 30±83 | 51±83 | 0.006 |
| uCreatinine, mM | 11.4±7.2 | 13.1±8.6 | 0.021 |
| Plasma metabolites | | | |
| pCreatinine, μM | 86±34 | 70±13 | 0.000 |
| pHcy, μM | 7.4±3.3 | 6.0±2.4 | 0.000 |
| pCys, μM | 263±61 | 228±68 | 0.000 |
| pCysGly, μM | 17.3±9.0 | 19.7±11.8 | 0.016 |
| pGSH, μM | 3.3±1.8 | 4.2±3.1 | 0.000 |
| pMet, μM | 41±18 | 42±19 | 0.429 |
| Anti-*N*-Hcy, A_492_ | 0.095±0.126 | 0.074±0.087 | 0.039 |
|  |  |  |  |
| Plasma glucose, mM | 6.5±2.7 | 5.6±0.7 | 0.000 |
| Total cholesterol, mM | 179±49 | 208±40 | 0.000 |
| LDL cholesterol, mM | 104±42 | 119±37 | 0.000 |
| HDL cholesterol, mM | 52±28 | 66±18 | 0.000 |
| Triglycerides, mM | 132±68 | 114±79 | 0.006 |
| Established risk factors | | | |
| BMI, kg/m^2^ | 26.3±4.7 | NA |  |
| GFR, mL/min/1.73 m^2^ | 74.2±17.5 | 86.4±7.8 | 0.000 |
| Earlier CVD, % | 23.3 | 2.3 | 0.000 |
| Earlier MI #6, % | 8.7 | 0.7 | 0.000 |
| Other heart disease, % | 22.5 | 4.3 | 0.000 |
| Hypertension, % | 77.5 | 21.7 | 0.000 |
| Diabetes, % | 24.0 | 3.7 | 0.000 |
| Smoking, % | 47.5 | NA |  |
| Medications, % | 78.3 | 7.7 | 0.000 |
| Age, years | 68±12 | 50±17 | 0.000 |
| Female sex, % | 45.0 | 59.4 | 0.926 |
| GFR, glomerular filtration rate; BMI, body mass index; CVD, cardiovascular disease; HDL, high-density lipoprotein; LDL, low-density lipoprotein; Abs_max_, maximum absorbance at 335 nM; CLT, clot lysis time; Anti *N*-Hcy, anti-*N*-Hcy-protein autoantibodies; NA, not available. Urinary and plasma metabolites are shown by a letter ‘u’ or ‘p’, respectively, preceding the metabolite’s name. Hcy, homocysteine; HTL, Hcy-thiolactone; Cys, cysteine; CysGly, cysteinylglycine’ Met, methionine. | | | |

**Table S2.** **Pearson correlation coefficients for relationships between turbidimetric clotting and lysis variables in stroke patients and healthy individuals.** Nomenclature is after Carter *et al*., *Arterioscler Thromb Vasc Biol* 2007; 27:2783-2789. The clotting and lysis variables are illustrated in **Figure S1**.

* Terms Abs_max_ and CLT, referring to terms MaxAbs and Lysis50_MA_, respectively, of Carter *el al*. ATVB 2007, have been use in the present study. *P* values for these correlations were <0.000 except where written otherwise.

|  | **Clotting/lysis correlation coefficients** | | | | | | |
| --- | --- | --- | --- | --- | --- | --- | --- |
| Variable | MaxAbs, Abs_max_* | Clot Rate | Lys50_t0_ | Lys50_tlag_ | Lysis50_MA_, CLT* | Lysis Rate | Lysis Area  AUC |
|  | **Stroke patients** (n = 191) | | | | | | |
| Lag | -0.61 | -0.65 | 0.45 | -0.02  (*P*=0.801) | -0.01 (*P*=0.929) | 0.44 | 0.41 |
| AbsMax,  Abs_max_* |  | 0.89 | -0.10  (*P*=0.183) | 0.21  (*P*=0.003) | **0.29** | -0.77 | 0.88 |
| Clot Rate |  |  | -0.45 | -0.16  (*P*=0.026) | 0.01  (*P*=0.837) | -0.72 | 0.61 |
| Lys50_t0_ |  |  |  | 0.89 | 0.86 | -0.07  (*P*=0.204) | 0.65 |
| Lys50_tlag_ |  |  |  |  | 0.75 | -0.05  (*P*=0.553) | 0.51 |
| Lysis50_MA_, CLT* |  |  |  |  |  | -0.05  (*P*=0.527) | 0.58 |
| Lysis Rate |  |  |  |  |  |  | -0.54 |
|  |  |  |  |  |  |  |  |
|  | **Healthy controls** (n = 291) | | | | | | |
| Lag | -0.60 | -0.51 | 0.17  (*P*=0.003) | -0.29 | -0.14 (*P*=0.013) | 0.44 | 0.42 |
| AbsMax,  Abs_max_* |  | 0.88 | -0.32 | 0.60 | 0.55 | -0.70 | 0.88 |
| Clot Rate |  |  | 0.00  (*P*=0.982) | 0.25 | 0.35 | -0.70 | 0.64 |
| Lys50_t0_ |  |  |  | 0.89 | 0.86 | -0.07  (*P*=0.204) | 0.65 |
| Lys50_tlag_ |  |  |  |  | 0.91 | -0.27 | 0.82 |
| Lysis50_MA_, CLT* |  |  |  |  |  | -0.28 | 0.79 |
| Lysis Rate |  |  |  |  |  |  | -0.48 |

| **Table S3**. *P* values for Pearsons’s correlations of fibrin clot properties *vs*. plasma sulfur-containing amino acid metabolites and creatinine in stroke patients and healthy individuals. | | | | | | | | |
| --- | --- | --- | --- | --- | --- | --- | --- | --- |
| Variable | Fibrin clot properties | | Sulfur-containing amino acid metabolites | | | | | pCreatinine |
|  | CLT | Abs_max_ | pHcy | pCys | pCysGly | pGSH | pMet |  |
| Healthy individuals, *P* values | | | | | | | | |
| CLT |  | 0.000(+) | ns | ns | ns | ns | ns | ns |
| Abs_max_ | 0.000(+) |  | ns | ns | ns | ns | ns | ns |
| pHcy | ns | ns |  | 0.000(+) | 0.000(+) | 0.000(+) | ns | 0.000(+) |
| pCys | ns | ns |  |  | 0.000(+) | 0.000(+) | ns | ns |
| pCysGly | ns | ns |  |  |  | 0.000(+) | ns | 0.008(+) |
| pGSH | ns | ns |  |  |  |  | ns | ns |
| pMet | ns | ns |  |  |  |  |  | ns |
| Stroke patients, *P* values | | | | | | | | |
| CLT |  | 0.000(+) | ns | ns | ns | 0.021(-) | ns | ns |
| Abs_max_ | 0.000(+) |  | ns | ns | ns | ns | 0.009(-) | ns |
| pHcy | ns | ns |  | 0.000(+) | 0.000(+) | 0.000(+) | ns | 0.035(+) |
| pCys | ns | ns |  |  | 0.000(+) | 0.000(+) | ns | 0.040(+) |
| pCysGly | 0.057(-) | ns |  |  |  | 0.000(+) | ns | ns |
| pGSH | 0.021(-) | ns |  |  |  |  | ns | ns |
| pMet | ns | 0.009(+) |  |  |  |  |  | ns |
|  | * *P* values for associations affected by stroke are highlighted in bold. Symbols (-) and (+) show negative and positive correlations, respectively. CLT, clot lysis time; Abs_max_, maximum absorbance at 335 nm. | | | | | | | |

| **Table S4.** Contribution of urinary and plasma sulfur-containing amino acid metabolites to the risk of ischemic stroke*. | | | | | |
| --- | --- | --- | --- | --- | --- |
|  | Cox & Snell R^2^ | Risk of stroke, % | Nagelkerke R^2^ | Risk of stroke, % | Avg risk of stroke, % |
| Model 3 | 53 |  | 71 |  |  |
| -uHTL | 52 | 1 | 70 | 1 | 1 |
| -uHcy | 52 | 1 | 71 | 0 | 0.5 |
| -uCys | 52 | 1 | 71 | 0 | 0.5 |
| **-uGSH** | 51 | 2 | 70 | 1 | 1.5 |
| -uHTL, -uHcy, -uCys, -uGSH | 47 | 6 | 64 | 7 | **6.5** |
| -pHcy | 53 | 0 | 71 | 0 | 0 |
| **-pCys** | 51 | 2 | 70 | 1 | 1.5 |
| -pCysGly | 52 | 1 | 71 | 0 | 0.5 |
| -pGSH | 53 | 0 | 71 | 0 | 0 |
| -pHcy, -pCys, -pCysGly,  -pGSH | 51 | 2 | 70 | 1 | **2** |
| -uHTL, -uHcy, -uCys, -uGSH,  -pHcy, -pCys, -pCysGly,  -pGSH | 45 | 8 | 61 | 10 | **9** |
|  |  |  |  |  |  |
| Model 1 | 48 |  | 65 |  |  |
| -uHTL | 47 | 1 | 64 | 1 | 1 |
| -uHcy | 48 | 0 | 65 | 0 | 0 |
| -uCys | 47 | 1 | 64 | 1 | 1 |
| -uCysGly | 48 | 0 | 65 | 0 | 0 |
| -uGSH | 47 | 1 | 64 | 1 | 1 |
| -uHTL, -uHcy, -uCys,  -uCysGly, -uGSH | 40 | 8 | 54 | 9 | **8.5** |
| -pHcy | 48 | 0 | 65 | 0 | 0 |
| **-pCys** | 46 | 2 | 63 | 2 | 2 |
| -pCysGly | 47 | 1 | 64 | 1 | 1 |
| -pGSH | 48 | 0 | 65 | 0 | 0 |
| -pHcy, -pCys, -pCysGly,  -pGSH | 46 | 2 | 62 | 3 | **2.5** |
| -uHTL, -uHcy, -uCys, -uGSH,  -pHcy, -pCys, -pCysGly,  -pGSH | 36 | 12 | 49 | 16 | **14** |
| * Risk of stroke was calculated by subtracting an R^2^ value for a model w/o shown metabolite from R^2^ value for Model 1 or 3. CLT, clot lysis time; Abs_max_, maximum absorbance at 335 nM. | | | | | |
